# Phase separation of insulin receptor substrate 1 drives the formation of insulin/IGF-1 signalosomes

**DOI:** 10.1038/s41421-022-00426-x

**Published:** 2022-06-28

**Authors:** Xiu Kui Gao, Xi Sheng Rao, Xiao Xia Cong, Zu Kang Sheng, Yu Ting Sun, Shui Bo Xu, Jian Feng Wang, Yong Heng Liang, Lin Rong Lu, Hongwei Ouyang, Huiqing Ge, Jian-sheng Guo, Hang-jun Wu, Qi Ming Sun, Hao-bo Wu, Zhang Bao, Li Ling Zheng, Yi Ting Zhou

**Affiliations:** 1 Department of Biochemistry and Department of Orthopaedic Surgery of the Second Affiliated Hospital, Zhejiang University School of Medicine, Hangzhou, Zhejiang China; 2 Dr. Li Dak Sum & Yip Yio Chin Center for Stem Cell and Regenerative Medicine, Zhejiang Provincial Key Lab for Tissue Engineering and Regenerative Medicine, Zhejiang University School of Medicine, Hangzhou, Zhejiang China; 3 ZJU-UoE Institute, Zhejiang University School of Medicine, Hangzhou, Zhejiang China; 4 Department of Respiratory Medicine, the First Affiliated Hospital, Zhejiang University School of Medicine, Hangzhou, Zhejiang China; 5 College of Life Sciences, Key Laboratory of Agricultural Environmental Microbiology of Ministry of Agriculture, Nanjing Agricultural University, Nanjing, Jiangsu China; 6 Department of Immunology, Zhejiang University School of Medicine, Hangzhou, Zhejiang China; 7 Department of Respiratory Care, Regional Medical Center for the National Institute of Respiratory Diseases, Sir Run Run Shaw Hospital, Zhejiang University School of Medicine, Hangzhou, Zhejiang China; 8 Department of Pathology of Sir Run Run Shaw Hospital, Center of Cryo-Electron Microscopy, Zhejiang University School of Medicine, Hangzhou, Zhejiang China; 9 Department of Biochemistry and Department of General Intensive Care Unit of the Second Affiliated Hospital, Zhejiang University School of Medicine, Hangzhou, Zhejiang China

**Keywords:** Insulin signalling, Cell growth

## Abstract

As a critical node for insulin/IGF signaling, insulin receptor substrate 1 (IRS-1) is essential for metabolic regulation. A long and unstructured C-terminal region of IRS-1 recruits downstream effectors for promoting insulin/IGF signals. However, the underlying molecular basis for this remains elusive. Here, we found that the C-terminus of IRS-1 undergoes liquid-liquid phase separation (LLPS). Both electrostatic and hydrophobic interactions were seen to drive IRS-1 LLPS. Self-association of IRS-1, which was mainly mediated by the 301–600 region, drives IRS-1 LLPS to form insulin/IGF-1 signalosomes. Moreover, tyrosine residues of YXXM motifs, which recruit downstream effectors, also contributed to IRS-1 self-association and LLPS. Impairment of IRS-1 LLPS attenuated its positive effects on insulin/IGF-1 signaling. The metabolic disease-associated G972R mutation impaired the self-association and LLPS of IRS-1. Our findings delineate a mechanism in which LLPS of IRS-1-mediated signalosomes serves as an organizing center for insulin/IGF-1 signaling and implicate the role of aberrant IRS-1 LLPS in metabolic diseases.

## Introduction

In addition to the classic organelles that are surrounded by lipid membranes, recent evidence indicates that non-membrane-bound organelles that are driven by phase separation are also essential for controlling various cellular processes^1–8^. These membraneless structures behave as liquid droplets in the cytoplasm or nucleoplasm. In these structures, either multiple modular interaction domains or intrinsically disordered regions (IDRs) of proteins can mediate the inter- or intra-molecular interactions underlying their liquid-like molecular condensations^9,10^. In addition to maintaining specific organelle structures, phase separation enables hub proteins to assemble signalosomes which promote the speed of signaling outputs^11,12^. Both Wnt signaling and T cell receptor (TCR) signaling, to give two examples, have been demonstrated to rely on essential adaptor protein-mediated phase separation^13–17^. However, the roles of phase separation in the regulation of many other signaling pathways await further exploration.

Insulin and insulin-like growth factors (IGFs) elicit a variety of pivotal physiological events, including those related to metabolism, differentiation, or growth^18–22^. Insulin receptor substrate (IRS) proteins play essential roles in mediating insulin/IGF signaling by recruiting a series of downstream effectors^23^. As the first identified substrate of IR/IGFR, knockout of IRS-1 led to metabolic defects, growth retardation, and β cell hyperplasia^24,25^. In humans, polymorphisms of IRS-1 have been found to be associated with type 2 diabetes and obesity^26,27^. IRS-1 contains no transmembrane domain but an amino-terminal pleckstrin homology (PH) and a phosphotyrosine-binding domain (PTB), followed by a carboxyl-terminal region which is enriched in Ser and Thr residues^28^. Interestingly, although the C-terminus of IRS-1 is essential for modulating the activation and stability of IRS-1, it is largely unstructured^29^. Moreover, IRS-1 was found to form high-molecular-mass complexes in different types of cells^30^. Since these intrinsically disordered regions, which are conformationally dynamic and do not adopt stable secondary or tertiary structures, are often essential for mediating the phase transition of proteins^9^, it is therefore of interest to consider if the C-terminus of IRS-1 is involved in phase separation and to further delineate such implications upon insulin/IGF signaling.

Here, we demonstrate that the C-terminus of IRS-1 undergoes phase separation. IGF-1 stimulation enhanced IRS-1 phase separation which then recruited downstream effectors to form insulin/IGF signalosomes. We further identified that the 301–600 residues, as a self-association region (SAR), is essential to the formation of IRS-1 droplets and the transduction of insulin/IGF signaling. Importantly, metabolic disease-derived G972R mutation results in a reduced ability of LLPS, potentially implicating the involvement of aberrant IRS-1 phase separation in various metabolic disorders.

## Results

### IRS-1 forms liquid droplets in cells via its intrinsically disordered region

**Fig. 1:**
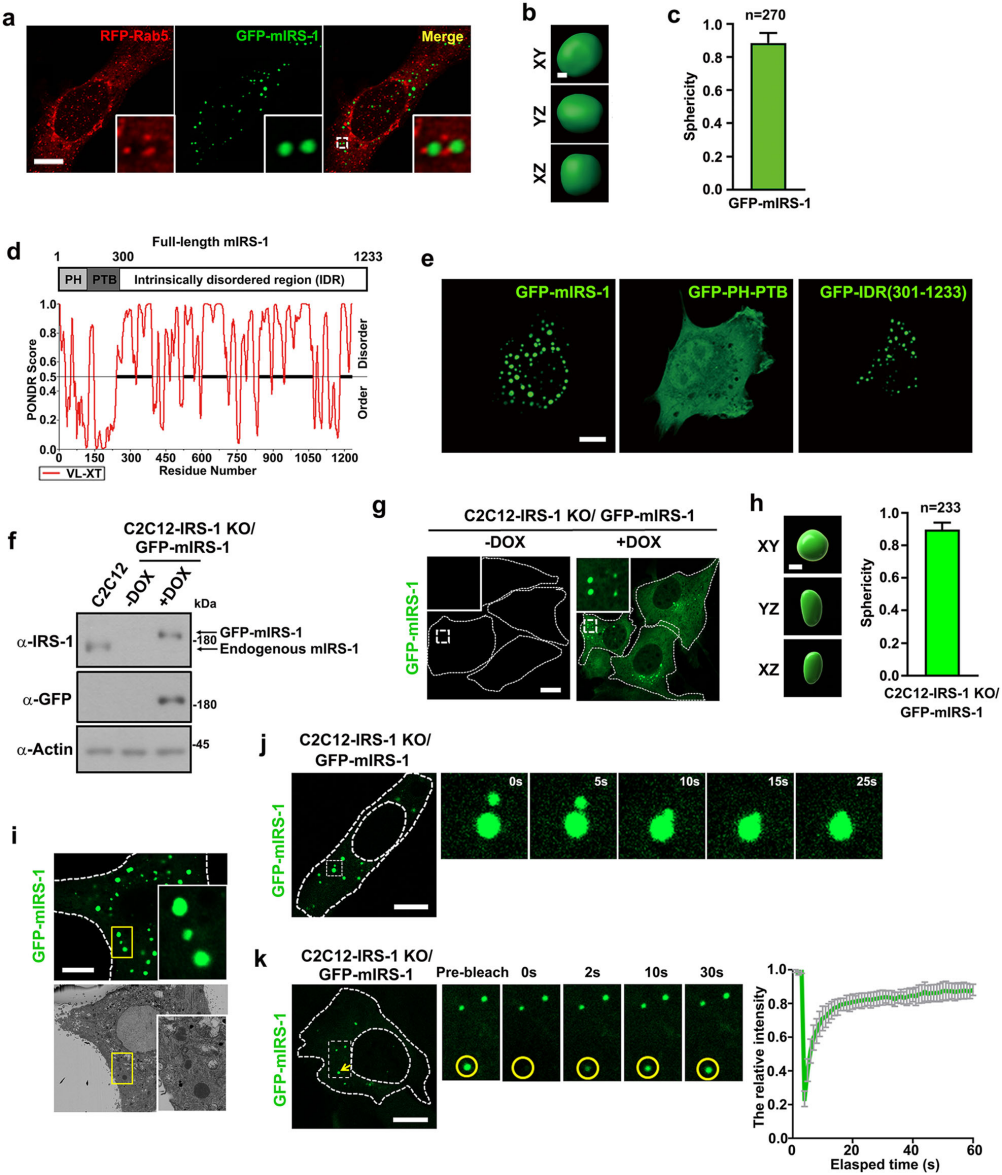
IRS-1 forms liquid-like droplets in cells. **a** Confocal images of representative C2C12 myoblasts co-expressing RFP-Rab5 and GFP-mIRS-1. Scale bar, 10 µm. **b** Rendered 3D shapes of an mIRS-1 droplet. The panels show the XY, XZ, and YZ planes. Scale bar, 1 µm. **c** A plot showing the sphericity of mIRS-1 droplets (*n* = 270). The quantification result is shown as mean ± SEM. **d** Protein sequence and disorder prediction (PONDR) of the mIRS-1 [1233 amino acids (aa)]. **e** Representative images of mIRS-1 droplets in C2C12 cells expressing either GFP-tagged mIRS-1, PH-PTB region (1-300 amino acids), or mIRS-1 IDR (301–1233 amino acids). Scale bar, 10 µm. **f** Immunoblot analysis of IRS-1 expression levels in C2C12 wildtype, uninduced or DOX-induced C2C12-IRS-1 KO/GFP-mIRS-1 cell line. **g** Confocal images of GFP-mIRS-1 in uninduced or DOX-induced C2C12-IRS-1 KO/GFP-mIRS-1 cells. **h** Rendered 3D shapes of an mIRS-1 droplet in DOX-induced C2C12-IRS-1 KO/GFP-mIRS-1 cells and a plot showing the sphericity of mIRS-1 droplets (*n* = 233). Scale bar, 1 µm. Data are shown as mean ± SEM. **i** Correlative light-electron microscopy (CL-EM) of C2C12 cells transiently transfected with GFP-mIRS-1. Scale bar, 10 µm. The upper panel insert shows three GFP-mIRS-1 foci and the lower panel inserts show three membrane-less mIRS-1 bodies. **j** Time-lapse imaging showing fusion of two mIRS-1 droplets in DOX-induced C2C12-IRS-1 KO/GFP-mIRS-1 cells. Scale bar, 10 µm. **k** Confocal images and quantification of mIRS-1 fluorescence recovery after photobleaching in DOX-induced C2C12-IRS-1 KO/GFP-mIRS-1 cells. Scale bar, 10 µm. Right panel: quantification of fluorescence intensity recovery of photobleached IRS-1 bodies (*n* = 11). Data are shown as means ± SD.

Our recent study discovered that Rab5, an essential modulator of endosomes, regulated the activation of IRS-1 by coordinating its intracellular membrane localization^31^. Here, exogenously expressed IRS-1 displayed spherical structures which demonstrated occasional attachments to Rab5-positive puncta in either mouse (mIRS-1) or human (hIRS-1) contexts (Fig. 1a–c; Supplementary Fig. S1a–c). The spherical pattern of IRS-1 raised the hypothesis that IRS-1 may undergo phase transition. We thus analyzed IRS-1 sequences using the PONDR program which indicated the C-terminus ends of mIRS-1 and hIRS-1 as intrinsically disordered regions (IDRs) (Fig. 1d; Supplementary Fig. S1d). The C-terminus of IRS-2, another IRS family member, was also predicted to be an IDR (Supplementary Fig. S1e). We further examined several cell lines expressing GFP-tagged IRS-1, especially the N-terminal 1–300 region containing PH and PTB domains or the C-terminus (301–1233). We found that both the full-length and the C-terminus of mIRS-1 formed droplet-like spheres in the cytosol of C2C12 myoblast, 293 T cells, or MCF-7 Cells (Fig. 1e; Supplementary Fig. S1f, g). Exogenously expressed GFP-mIRS-2, which also acts as a scaffold protein in insulin/IGF signaling^23,29^, displayed spherical foci in cells (Supplementary Fig. S1h). We co-expressed FLAG-mIRS-1 and GFP-mIRS-1 in cells and found that the FLAG-mIRS-1 droplets detected by antibodies seem hollow, while the GFP-mIRS-1 detected by GFP fluorescence appear as solid (Supplementary Fig. S1i). This observation is similar to the previously reported Dvl2 and TDP-43 puncta formed by phase separation^32,33^. Such a hollowness is likely due to the lack of antibody access to the center of such kinds of droplets^32,33^.

To exclude the possibility that the formation of IRS-1 puncta was due to high levels of overexpressed protein, we first established an IRS-1 knockout (KO) C2C12 cell line (C2C12-IRS-1 KO) (Supplementary Fig. S1j, k). Notably, endogenous IRS-1 puncta could be observed in C2C12 myoblasts (Supplementary Fig. S1k, left panel), as well as in differentiated myotubes (Supplementary Fig. S1l), but not in the C2C12-IRS-1 KO cells (Supplementary Fig. S1k, right panel). We then used the IRS-1 KO cells to set up a stable cell line (C2C12-IRS-1 KO/GFP-mIRS-1) which expressed GFP-mIRS-1 in an inducible manner. Since we plan to perform fluorescence recovery after the photobleaching (FRAP) and foci fusion analysis to examine the liquid property of mIRS-1 foci, GFP was chosen as the tag for mIRS-1. By titrating the inducer (Doxycycline, DOX) concentration and the induction time, we were able to express exogenous GFP-mIRS-1 protein close to endogenous levels in parental C2C12 myoblasts (Fig. 1f). Under these optimized conditions, cells display similar expression levels of GFP-mIRS-1 (Supplementary Fig. S1m) and we therefore confirmed the formation of IRS-1 spherical structures in cells (Fig. 1g, h).

To further characterize the IRS-1 droplets, electron microscopy (EM) coupled with correlative confocal imaging analysis was performed. EM revealed mIRS-1 spheres as lacking a membrane, and we noted this as defining feature of phase-separated biomolecular condensates and membrane-less organelles (Fig. 1i). Moreover, live imaging analysis showed that IRS-1 droplets in C2C12-IRS-1 KO/GFP-mIRS-1 cells could undergo fusion (Fig. 1j). This suggested liquid-like properties for these IRS-1 spherical structures. FRAP of GFP-mIRS-1 droplets disclosed a fast recovery rate in C2C12-IRS-1 KO/GFP-mIRS-1 myoblasts (Fig. 1k). Again, this indicated IRS-1 droplets to be highly dynamic and to readily exchange molecules with the surrounding cytosol. Moreover, mIRS-2 foci also displayed fast recovery rate in FRAP assay and underwent fusion (Supplementary Fig. S1n, o).

### IRS-1 undergoes liquid-liquid phase separation in vitro

**Fig. 2:**
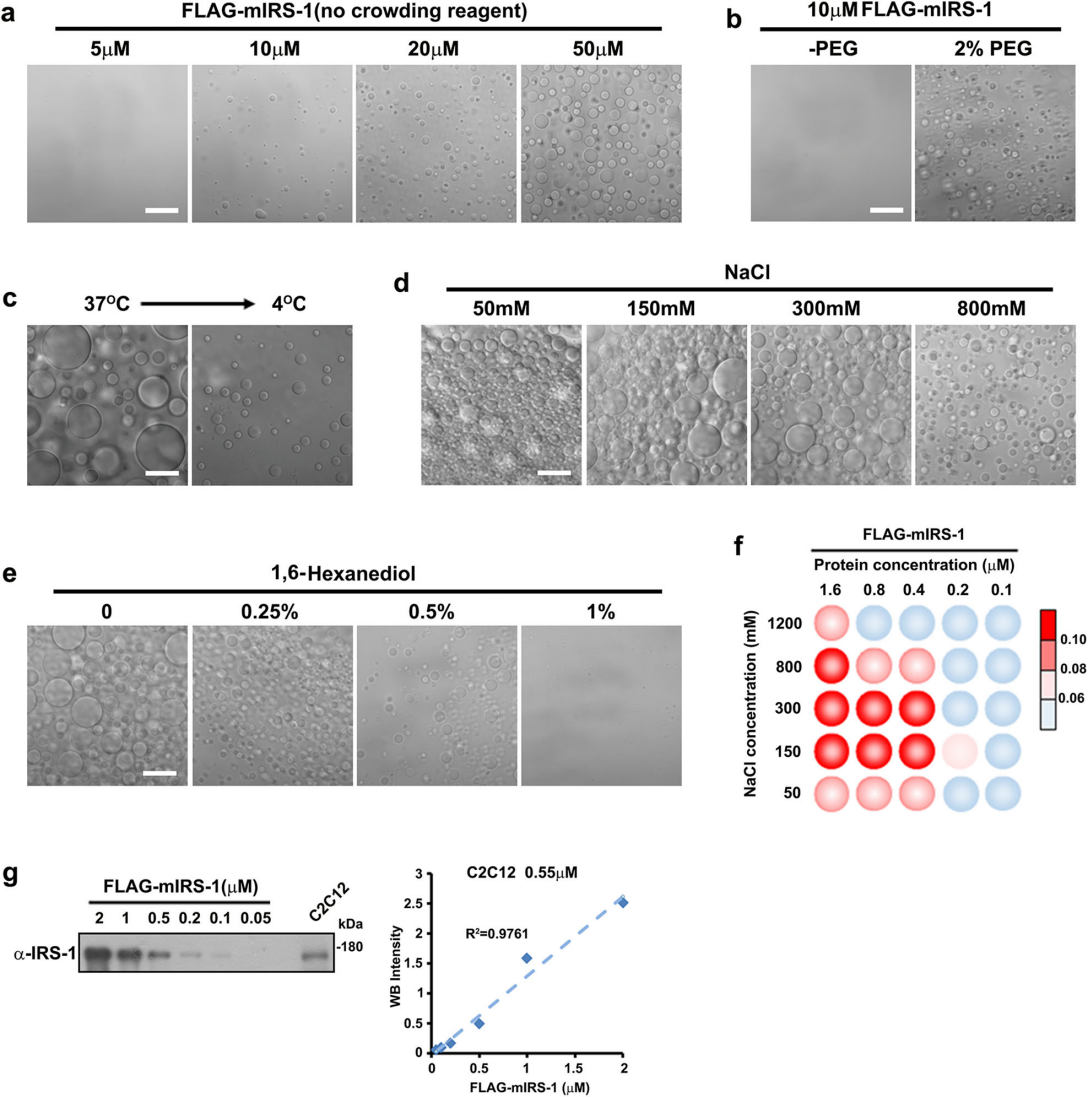
IRS-1 undergoes phase separation in vitro. **a** DIC images of FLAG-mIRS-1 LLPS at a series of protein concentrations (5–50 μM) (50 mM Tris-HCl pH 7.5, 10% glycerol, 1 mM DTT, 150 mM NaCl). The proteins were incubated with phase separation buffer at room temperature for 40 min. Scale bar, 20 µm. **b** DIC images of FLAG-mIRS-1 LLPS in the presence of molecular crowding (2% w/v PEG-8000) (right). No phase separation was observed without crowding agent (left). The proteins were incubated with phase separation buffer at room temperature for 5 min. Scale bar, 20 µm. **c** LLPS of mIRS-1 at 37 °C or 4 °C for 10 min. Scale bar, 20 µm. **d** DIC images of FLAG-mIRS-1 LLPS under different salinity, as indicated. The proteins (10 μM) were incubated with phase separation buffer at room temperature for 20 min. Scale bar, 20 µm. **e** DIC images of FLAG-mIRS-1 LLPS with the addition of 1,6-hexanediol at indicated concentration. The proteins (10 μM) were incubated with phase separation buffer at room temperature for 20 min. Scale bar, 20 µm. **f** Phase diagrams of GFP-mIRS-1 with concentrations ranging from 0.1-1.6 μM in 50 mM Tris (pH 7.5), 2% (w/v) PEG-8000, and sodium chloride (ranging from 50–1200 mM). Blue dots indicate no phase separation. Red dots indicate phase separation. The LLPS ability of mIRS-1 under different conditions was color-coded on the basis of droplet turbidity as measured at OD600 when the proteins had been incubated with phase separation buffer at 37 °C for 120 min. **g** Quantification result of endogenous IRS-1 protein concentrations in C2C12 cells based on immunoblot densitometry analysis performed on cell lysates and purified FLAG-mIRS-1 protein.

To test whether IRS-1 could undergo phase separation in vitro, we purified recombinant FLAG-mIRS-1 to perform differential interference contrast (DIC) microscopy analysis (Supplementary Fig. S2a). Figure 2a shows DIC micrographs of a 37 °C solution of mIRS-1 at a range of concentrations. The solution was clear at the start of the imaging process and droplet formation was then observed after 40 minutes at a concentration of 10 µM (Fig. 2a). Increased protein concentrations led to increased numbers and larger sizes of mIRS-1 spheres (Fig. 2a). Polyethylenglycol (PEG) was then used to mimic intracellular crowding conditions^34,35^. We found that a low concentration of PEG (2%) was enough to accelerate the formation of mIRS-1 droplets (Fig. 2b). A higher PEG concentration could further induce increasing numbers and larger droplets (Supplementary Fig. S2b). Moreover, the mIRS-1 droplets could be observed when incubated at 37 °C, but were much reduced at 4 °C (Fig. 2c). Increasing salinity also reduced mIRS-1 droplet formation (Fig. 2d) and the addition of 1,6-hexanediol, an aliphatic alcohol that weakens hydrophobic interactions, similarly suppressed the assembly of mIRS-1 droplets (Fig. 2e). Furthermore, mIRS-1 droplet formation was not disrupted by either RNase A or DNase, indicating the phase separation of IRS-1 to be independent to nucleic acids (Supplementary Fig. S2c). It was concluded that IRS-1 phase separation seems to be driven by both electrostatic and hydrophobic interactions.

In addition, we established the phase separation diagrams of mIRS-1 by performing droplet formation assays of various concentrations of sodium chloride and mIRS-1^36^. We observed that in the presence of 150 mM NaCl, a level close to the physiological salt concentration in cells^37^, mIRS-1 was able to undergo LLPS from concentrations of 0.2 µM or above (Fig. 2f). We then measured the protein concentrations of endogenous IRS-1 in various cell lines. The endogenous concentration of IRS-1 protein in cells was approximately 0.2 to 0.7 µM (0.28 µM in MCF7, 0.55 µM in C2C12, 0.62 µM in 293 T) (Fig. 2g; Supplementary Fig. S2d), which was close to the critical concentration (0.2 µM) of mIRS-1 required for LLPS in vitro (Fig. 2f). We further performed FRAP assay and detected fusion process using purified mIRS-1. Upon contact, the iFluor 488-labled mIRS-1 droplets coalesced into larger ones in vitro (Supplementary Fig. S2e). After photobleaching, about 90% of mIRS-1 exchanged with the surrounding solution within 15 s (Supplementary Fig. S2f). Together, these results demonstrated that the purified IRS-1 undergoes LLPS in vitro.

### Self-association mediates the phase separation of IRS-1

The assembly of phase separation-mediated signalosomes relies on the polymerization of hub proteins^12^. Purified IRS-1 forms liquid-like droplets (Fig. 2), indicating that the self-association-mediated polymerization of IRS-1 drives its phase separation. Since both electrostatic and hydrophobic interactions contribute to IRS-1 phase separation (Fig. 2d, e), we proposed that it was primarily the self-association of IRS-1 that may mediate its phase separation. We first tested this hypothesis by examining the effects of the PTP1B D181A mutant on the IRS-1 phase separation. PTP1B is a phosphorylase of IRS-1 and the PTP1B D181A mutant has been shown to sequester its substrates^38,39^. This is in agreement with our finding that, compared to the PTP1B wildtype, the D181A mutant displayed enhanced interaction with IRS-1 (Supplementary Fig. S3a), which subsequently impaired the self-association of IRS-1 (Supplementary Fig. S3b). Consistent to our hypothesis, the PTP1B D181A mutant suppressed the formation of IRS-1 puncta in cells (Supplementary Fig. S3c).

**Fig. 3:**
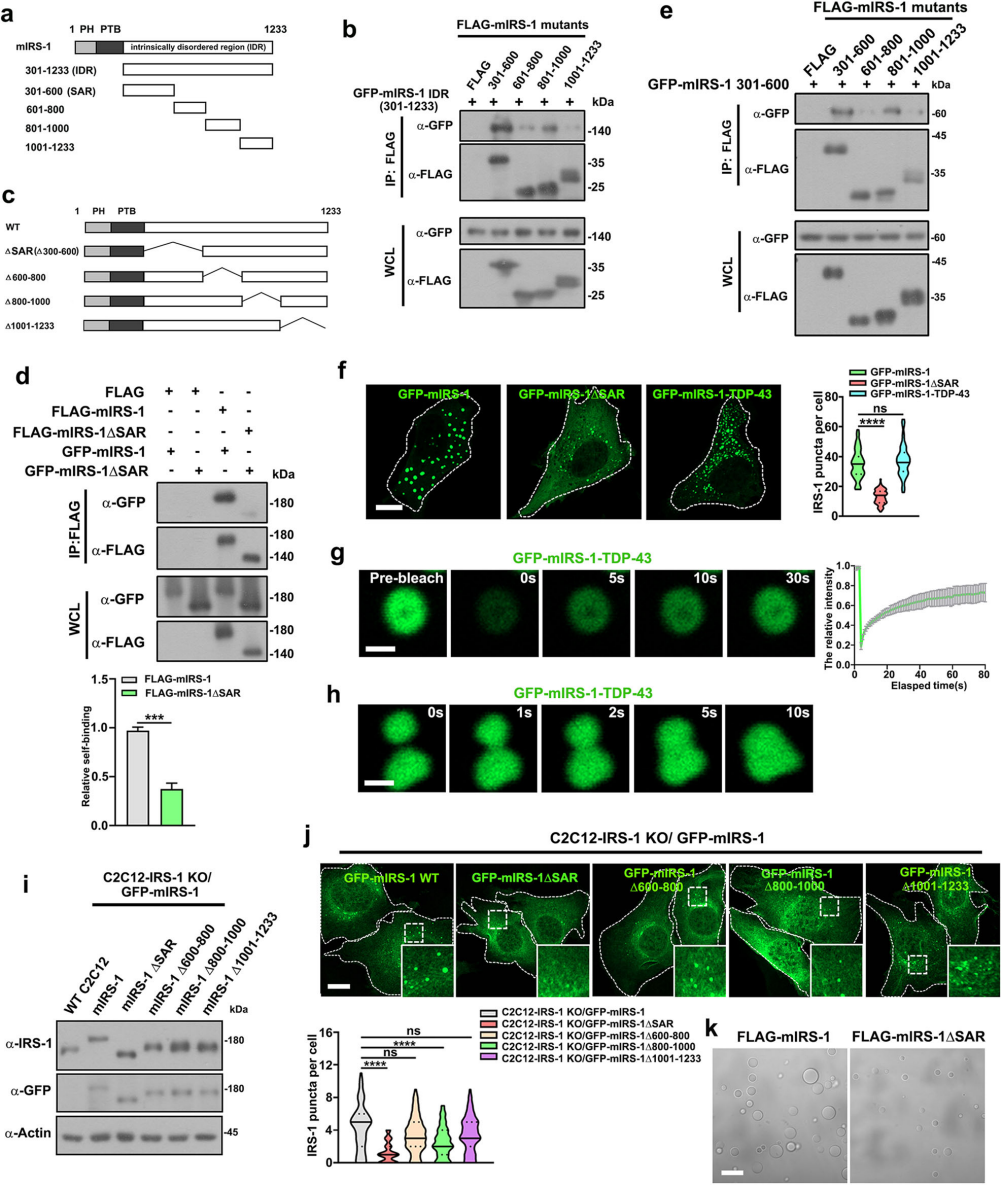
Phase separation of IRS-1 relies on self-association. **a** Schematic diagram of IDR of mIRS-1 and its truncation mutants. **b** FLAG-tagged mIRS-1 mutant constructs as shown in (**a**) were co-transfected with GFP-mIRS-1 IDR (301–1233) into 293 T cells for immunoprecipitation analysis. **c** Schematic diagram of mIRS-1 and its deletion mutants. **d** FLAG-tagged and GFP-tagged mIRS-1 or mIRS-1 ΔSAR mutants were co-transfected into 293 T cells for immunoprecipitation analysis. Data in the bar graphs represent the means ± SEM values of the ratios of densities for three independent experiments. ***p < 0.001. **e** FLAG-tagged mIRS-1 mutant constructs were co-transfected with GFP-mIRS-1 301–600 into 293 T cells for immunoprecipitation analysis. **f** Representative confocal images of GFP-tagged mIRS-1, mIRS-1 ΔSAR mutant, and mIRS-1-TDP-43 mutant in C2C12 cells. Quantification results of GFP-mIRS-1 and mutant puncta are shown as violin plots (*n* = 80). ****p < 0.0001. ns: not significant. **g** Confocal images and quantification of GFP-mIRS-1-TDP-43 mutant fluorescence recovery after photobleaching. Scale bar, 1 µm. **h** Time-lapse imaging showing fusion of two GFP-mIRS-1-TDP-43 droplets in cells. Scale bar, 1 µm. **i** Immunoblot analysis of the indicated proteins in C2C12-IRS-1 KO cell lines stably expressing GFP-mIRS-1 (mIRS-1 WT, mIRS-1 ΔSAR, mIRS-1 Δ600–800, mIRS-1 Δ800–1000, and mIRS-1 Δ1001–1233). **j** Confocal images of GFP-tagged mIRS-1 wildtype and mutants in C2C12 cell lines as indicated in (**i**). Scale bar, 10 µm. Quantification results of GFP-mIRS-1 or mutant puncta are shown as violin plots. ****p < 0.0001. ns not significant. **k** DIC images of FLAG-mIRS-1 and mIRS-1 ΔSAR LLPS. The proteins (1 μM) were incubated with phase separation buffer at room temperature for 20 min. Scale bar, 20 µm.

To further validate our hypothesis, we created a series of truncation mutants with the aim of identifying the essential regions of the mIRS-1 IDR responsible for mediating polymerization (Fig. 3a). Using a co-immunoprecipitation assay we showed that the 301–600 sequence acts as a major region to mediate a self-association interaction (Fig. 3b). To further verify if the 301–600 acts as a self-association region (SAR), a series of GFP-tagged mIRS-1 deletion or truncation mutants were created (Fig. 3c; Supplementary Fig. S3d, e). Deletion of the 300–600 region impaired the self-association of mIRS-1 (Fig. 3d). Notably, the 301–600 region interacts with not only itself, but also the 801–1000 region (Fig. 3e), indicating the presence of multivalent interactions in mIRS-1. Correspondingly, the exogenous expression of the Δ300-600 construct (hereafter designated IRS-1 ΔSAR) failed to form droplets in cells (Supplementary Fig. S3f, g). Similarly, the truncation mutants devoid of the SAR region (601–1233, 701–1233, 801–1233), also failed to form droplets (Supplementary Fig. S3h, i). We further performed detailed mapping by deleting 300–400, 400–500, and 500–600 (Supplementary Fig. S3j, k), respectively, and found that all the three regions are required for mediating the mIRS-1 phase separation in cells (Supplementary Fig. S3l). Furthermore, replacement of the SAR region with the LLPS-driving C-terminal domain (aa 267–414) of TDP43^17,40^ (hereafter designated mIRS-1-TDP-43) could restore droplet formation (Fig. 3f; Supplementary Fig. S3m). The mIRS-1-TDP-43 also displayed liquid-like properties (Fig. 3g, h).

Using the C2C12-IRS-1 KO cells (Supplementary Fig. S1j, k), we further established a series of stable cell lines which individually expressed the mIRS-1 deletion mutants, as shown in Fig. 3c, close to the endogenous IRS-1 levels (Fig. 3i). The foci formation was significantly reduced in the C2C12-IRS-1 KO/GFP-mIRS-1 ΔSAR cell line (Fig. 3j). Deletion of 800–1000 region, which also interacts with the SAR (Fig. 3e), also displayed mildly reduced puncta formation (Fig. 3j). In line with these findings, comparing to wildtype mIRS-1, purified FLAG-mIRS-1 ΔSAR mutants formed smaller and fewer droplets in vitro (Fig. 3k; Supplementary Fig. S3n), validating the essential roles of the SAR region in driving IRS-1 phase separation. These findings support the hypothesis that the polymerization of IRS-1, as mediated by self-association, is essential for its phase separation.

### Phase transition of IRS-1 is dynamically regulated by insulin/IGF signaling

**Fig. 4:**
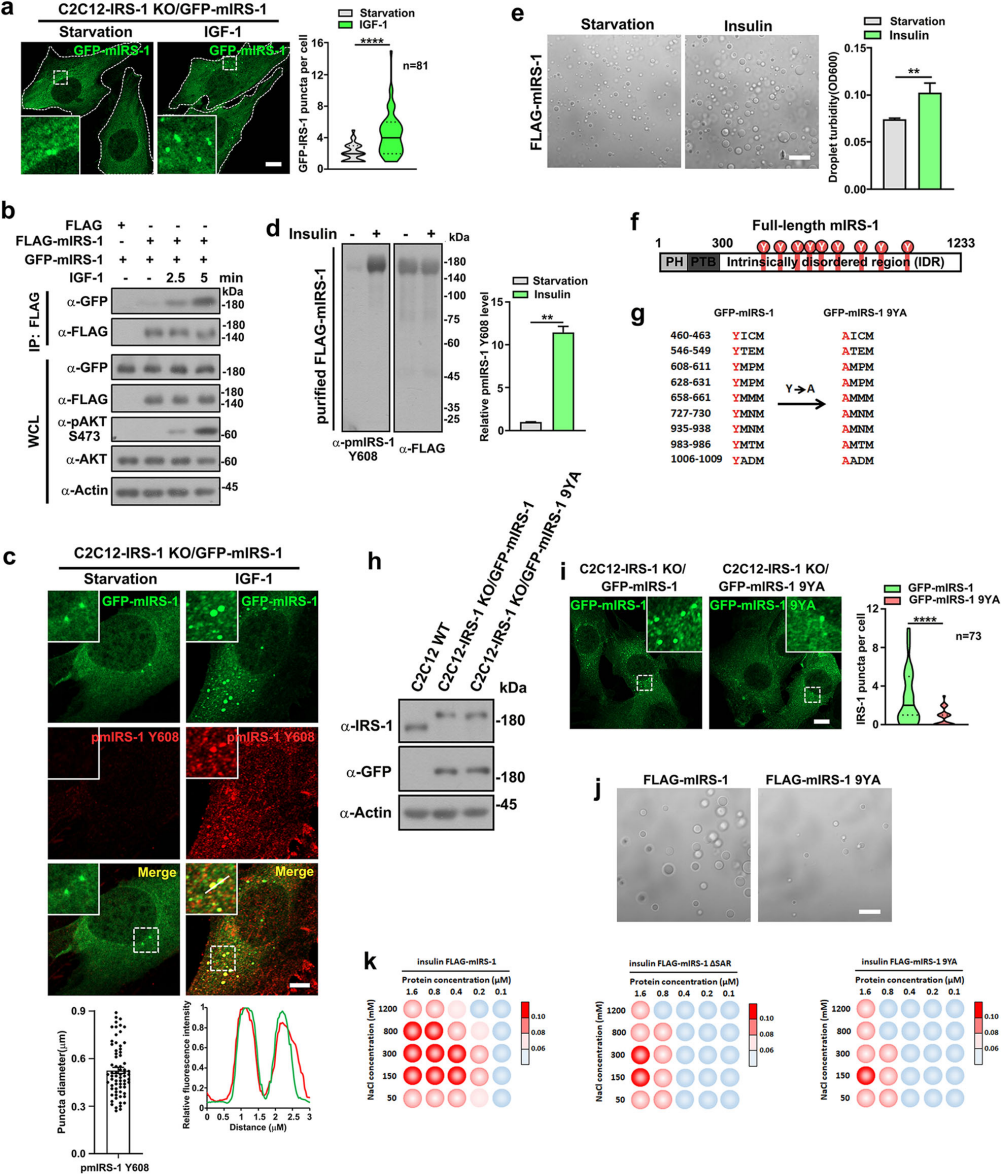
Insulin/IGF signal modulates the phase separation of IRS-1. **a** Confocal images of GFP-mIRS-1 foci in C2C12-IRS-1 KO/GFP-mIRS-1 cells treated with control or with IGF-1-conditioned (100 ng/mL) medium for 2.5 min. Scale bar, 10 µm. Quantitative analysis of number of mIRS-1 puncta is shown with results presented as violin plots. ***p < 0.001. **b** FLAG-tagged and GFP-tagged mIRS-1 were co-transfected into 293 T cells. Cells were serum starved for 16 h followed by IGF-1 stimulation and coimmunoprecipitation analysis. **c** Immunofluorescence staining of endogenous p-IRS-1 (Y608) antibody in C2C12-IRS-1 KO/GFP-mIRS-1 cells treated for 5 min with control medium or IGF-1-conditioned medium. Scale bar, 5 µm. Line scan showing the related intensity profiles of mIRS-1 with p-IRS-1 (Y608). The puncta diameter was quantified (*n* = 69). Data in the bar graphs represent the means ± SEM. **d** Immunoblot analysis of Y608 residue tyrosine phosphorylation of FLAG-mIRS-1 purified from starved or insulin-stimulated (15 min) 293 T cells. Quantification result is shown as means ± SEM. **p < 0.001. **e** DIC images of FLAG-mIRS-1 purified from starved or insulin-stimulated (15 min) 293 T cells. The proteins (1 μM) were incubated with phase separation buffer at room temperature for 20 min. Scale bar, 20 µm. Quantification result is shown as means ± SD. **p < 0.01. **f** Scheme indicating the location of the nine tyrosine residues of YXXM motifs in mIRS-1. **g** The nine tyrosine residues of the YXXM motifs in mIRS-1 were replaced by alanine residues. **h** Immunoblot analysis of the IRS-1 expression levels in C2C12 wildtype, C2C12-IRS-1 KO/GFP-mIRS-1, and C2C12-IRS-1 KO/GFP-mIRS-1 9YA cell lines. **i** Confocal images of GFP-tagged mIRS-1 or 9YA mutant in C2C12-IRS-1 KO/GFP-mIRS-1 or C2C12-IRS-1 KO/GFP-mIRS-1 9YA cell lines. Scale bar, 10 µm. Quantification results of GFP-mIRS-1 or 9YA puncta are shown and presented as violin plots. ****p < 0.0001. **j** DIC images of FLAG-mIRS-1 or FLAG-mIRS-1 9YA purified from 293 T cells. The proteins (1 μM) were incubated with phase separation buffer at room temperature for 20 min. Scale bar, 20 µm. **k** Phase diagrams of mIRS-1 wildtype, ΔSAR, and 9YA mutant proteins purified from insulin-stimulated cell lines in 50 mM Tris (pH 7.5), 2% (w/v) PEG-8000, and sodium chloride (ranging from 50–1200 mM).

Phase transition is emerging as an important concept in signal transduction^41,42^. It has been well characterized that the 301–1233 region of IRS-1 is peppered with tyrosine phosphorylation sites, which can be activated by IGF/insulin and subsequently function as interacting motifs for downstream Src homology 2 (SH2)-containing effectors^28,29^. This recognition raised the possibility that IRS-1 droplets could act as signalosomes for the insulin/IGF pathway. In accordance with the notion that dynamic assembly or disassembly upon signal activation is a hallmark of signalosomes^12^, we found that the number of IRS-1 puncta increased upon IGF-1 or insulin stimulation in C2C12-IRS-1 KO/GFP-mIRS-1 myoblasts (Fig. 4a; Supplementary Fig. S4a). Notably, the self-association of mIRS-1, which drives the IRS-1 phase separation, was enhanced upon IGF-1 or insulin stimulation (Fig. 4b; Supplementary Fig. S4b). We recently revealed that Rab5 activates IRS-1 to promote IGF signaling^31^. Dominant-negative form of Rab5 reduced the size of mIRS-1 droplets (Supplementary Fig. S4c) and promoted the recovery rate of mIRS-1 puncta (Supplementary Fig. S4d), indicating that inhibition of IRS-1 activation suppresses the LLPS.

We next carried out immunofluorescence using a phospho-specific antibody (Ab-pY612) that recognizes the phospho-tyrosine residue 608 of mIRS-1 (corresponding to the Y612 residue of hIRS-1) in IGF-1-stimulated C2C12-IRS-1 KO/GFP-mIRS-1 myoblasts. This tyrosine residue acts as the principal site for interaction with p85 and activation of PI3K^43^. Ab-pY608 immunoreactivity was rarely detectable in serum-starved myoblasts (Fig. 4c, left panel). By contrast, IGF-1 stimulation led to robust elevation of Ab-pY608, which colocalized well with the green GFP-mIRS-1 droplets (Fig. 4c). The sizes of Y608-positive mIRS-1 puncta were in the range of 0.3–1.1 µM (Fig. 4c). This finding indicates the involvement of tyrosine phosphorylation in IRS-1 phase transition. To validate this, we further purified FLAG-mIRS-1 proteins from serum starved or insulin/IGF-1-stimulated 293 T cells (Supplementary Fig. S4e). mIRS-1 purified from insulin- or IGF-1-stimulated 293 T cells, which displayed significantly higher levels of Y608 phosphorylation (Fig. 4d; Supplementary Fig. S4f), demonstrated a stronger ability of phase transition (Fig. 4e; Supplementary Fig. S4g). To further test the significance of the tyrosine residues of YXXM motifs to IRS-1 droplet formation, a construct was produced where the nine tyrosine residues were mutated to alanine (mIRS-1 9YA) (Fig. 4f, g). Ectopically expressed mIRS-1 9YA demonstrated a defective phase transition ability (Supplementary Fig. S4h). To ensure that this phenotype was not due to the differences in protein concentration, we further used the C2C12-IRS-1 KO cells to establish a cell line (C2C12-IRS-1 KO/GFP-mIRS-1 9YA) expressing the mIRS-1 9YA mutant at levels close to those of endogenous IRS-1 (Fig. 4h). It was notable that the droplet formation ability of mIRS-1 9YA was reduced compared to that of wild-type mIRS-1 in cells (Fig. 4i). Consistently, purified mIRS-1 9YA formed less and smaller foci in vitro (Fig. 4j; Supplementary Fig. S3n). We further purified mIRS-1 wildtype, ΔSAR, and 9YA mutant from insulin-stimulated cell lines to establish the phase separation diagrams. The critical concentration for LLPS of ΔSAR and 9YA mutant was much higher than that of the wildtype (Fig. 4k).

In line with our finding that the self-oligomerization mediates the IRS-1 phase separation, the mIRS-1 9YA mutant displayed a reduced self-association ability comparing to the wildtype mIRS-1 (Supplementary Fig. S4i). Consistently, replacing the tyrosine residues in 301–600 region (Y460 and Y546) or 801–1000 region (Y935 and Y983) also impaired the interaction between 301 and 600 and 801 and 1000 regions (Supplementary Fig. S4j, k). We thus concluded that the phase separation of IRS-1 is dynamically modulated by insulin/IGF signaling.

### IRS-1 droplets recruit downstream effectors to form insulin/IGF signalosomes

**Fig. 5:**
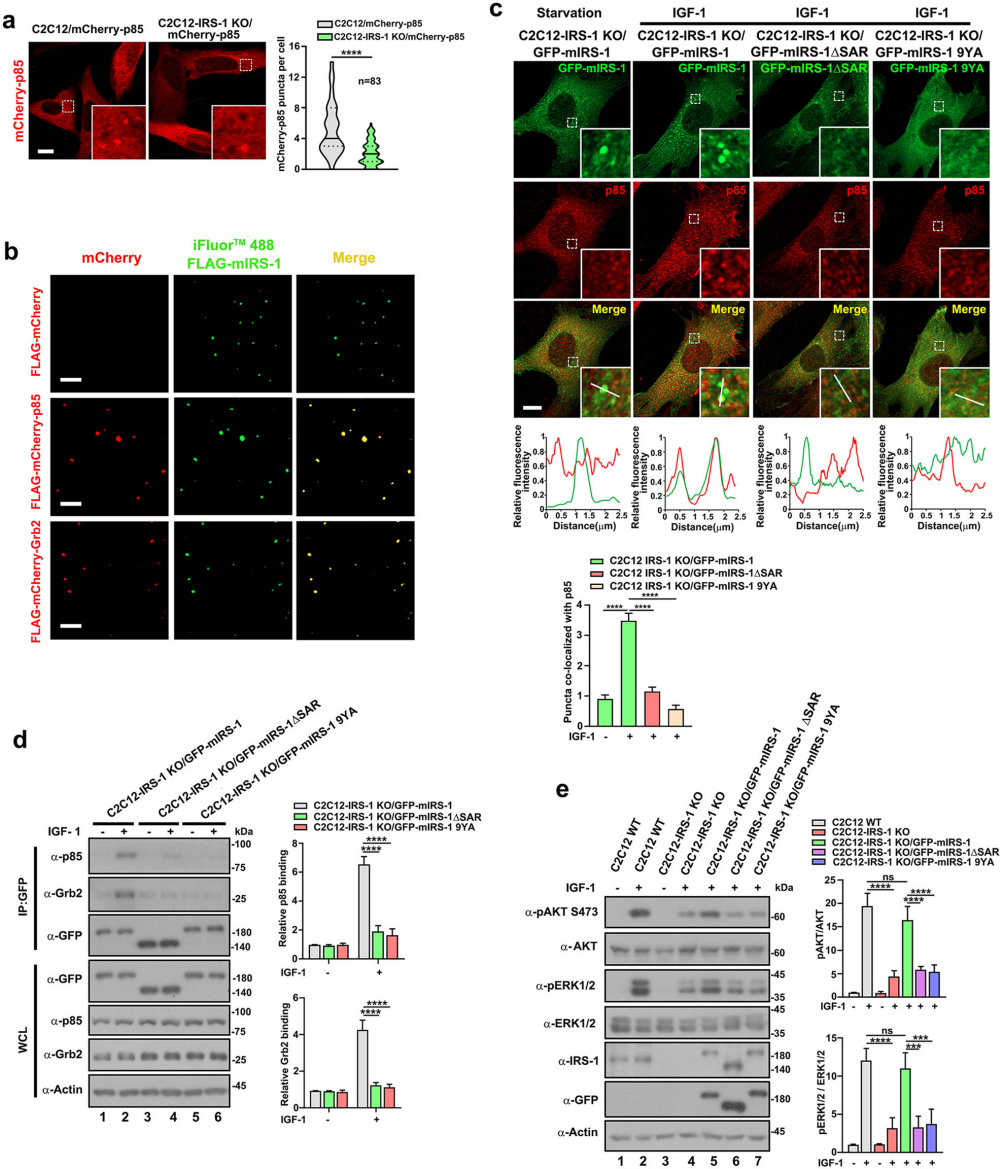
IRS-1 recruits downstream signaling molecules to form signalosomes. **a** Confocal image of representative C2C12 wildtype or C2C12-IRS-1 KO cells stably expressing mCherry-p85. Scale bar, 10 µm. Quantification results of mCherry-p85 puncta are shown and presented as violin plots. ****p < 0.0001. **b** Purified mCherry, mCherry-tagged p85 or Grb2 (1 μM) was incubated with iFluor™ 488-FLAG-mIRS-1 (1 μM) for 5 min. Scale bar, 20 µm. **c** Confocal images of endogenous p85 and GFP-mIRS-1 or mutants in the indicated cell lines. Scale bar, 10 µm. Line scan showing the related intensity profiles of mIRS-1 with p85. Scale bar, 10 µm. The GFP-positive puncta co-localized with p85 was quantified (*n* = 33). Data in the bar graphs represent the mean ± SEM. ****p < 0.0001. **d** GFP-tagged mIRS-1 wildtype or mutants were immunoprecipitated in IGF-1-stimulated or control C2C12-IRS-1 KO, C2C12-IRS-1 KO/GFP-mIRS-1, C2C12-IRS-1 KO/GFP-mIRS-1 ΔSAR or C2C12-IRS-1 KO/GFP-mIRS-1 9YA cell lines and then subjected to Western blot with p85 or Grb2 antibodies. Data in the bar graphs represent the mean ± SEM values of the ratios of densities for three independent experiments. ****p < 0.0001. **e** Immunoblot analysis of total and phosphorylated AKT and ERK levels in C2C12 wildtype, C2C12-IRS-1 KO, C2C12-IRS-1 KO/GFP-mIRS-1, C2C12-IRS-1 KO/GFP-mIRS-1 ΔSAR or C2C12-IRS-1 KO/GFP-mIRS-1 9YA cell lines treated with or without IGF-1 conditional medium for 2.5 min. Data in the bar graphs represent the means ± SEM values of the ratios of densities for three independent experiments. ***p < 0.001. ****p < 0.0001. ns not significant.

IRS-1 interacts with Grb2 or the p85 subunit of PI3K to regulate Ras-MAPK or AKT-mTOR signal pathways, respectively^19^. We next considered if the insulin/IGF signaling components could be recruited to IRS-1 droplets. To investigate this, we stably expressed mCherry-p85 in wildtype C2C12 (C2C12/mCherry-p85) or C2C12-IRS-1 KO (C2C12-IRS-1 KO/mcherry-p85) cells at levels close to those of endogenous p85 (Supplementary Fig. S5a). Though the majority of stably expressed mCherry-tagged p85 was seen to diffuse in the cytoplasm of C2C12/mCherry-p85 myoblasts, tiny foci of mCherry-p85 could still be observed (Fig. 5a, left panel). Notably, in comparison to C2C12/mCherry-p85 cells, the number of mCherry-p85 puncta was reduced in C2C12-IRS-1 KO/mcherry-p85 cells (Fig. 5a, right panel), indicating the foci formation of p85 to be dependent on IRS-1. Moreover, in C2C12/mCherry-p85 cells, both insulin and IGF-1 stimulation could enhance the foci formation of mCherry-p85 (Supplementary Fig. S5b, c). These findings indicate that insulin/IGF-1 signaling promotes the foci formation of p85, possibly through IRS-1. We thus hypothesized that the phase separation of IRS-1 is critical for recruiting downstream effectors of insulin/IGF signaling.

To verify this hypothesis, we first determined if p85 and Grb2 could be recruited by IRS-1 droplets in vitro. We thus mixed purified mCherry-tagged p85 or Grb2 with iFluor 488-labled mIRS-1 and found that mIRS-1 puncta recruited both downstream effectors in vitro (Fig. 5b). We next examined the cellular distribution of endogenous p85 or Grb2 in aforementioned engineered cell lines. The C2C12-IRS-1 KO/GFP-mIRS-1 9YA myoblasts were used as control cells since the mIRS-1 9YA mutant lacks the binding sites for p85 and Grb2. We found that upon IGF-1 stimulation, p85 formed distinct cytoplasmic puncta that colocalized with the IRS-1 droplets in C2C12-IRS-1 KO/GFP-mIRS-1 myoblasts (Fig. 5c). Contrastingly, p85 failed to show any such concentrations in either C2C12-IRS-1 KO/GFP-mIRS-1 ΔSAR or C2C12-IRS-1 KO/GFP-mIRS-1 9YA myoblasts (Fig. 5c). Similarly, endogenous Grb2 was recruited to IRS-1 droplets in C2C12-IRS-1 KO/GFP-mIRS-1 myoblasts (Supplementary Fig. S5d). The size of the puncta ranged from 0.3 to 0.9 µM (Supplementary Fig. S5e). In line with these observations, transiently expressed mIRS-1, but not the ΔSAR or the 9YA mutant, recruited mCherry-p85 or Grb2 (Supplementary Fig. S5f, g). Moreover, the mIRS-1-TDP-43 mutant, in which the SAR region was replaced by the LLPS-driving domain of TDP43 (Fig. 3f–h), also recruited both p85 and Grb2 (Supplementary Fig. S5h).

We further compared the interaction ability of mIRS-1, mIRS-1 ΔSAR, and mIRS-1 9YA with endogenous p85 or Grb2 using the C2C12-IRS-1 KO/GFP-mIRS-1, C2C12-IRS-1 KO/GFP-mIRS-1 ΔSAR, and C2C12-IRS-1 KO/GFP-mIRS-1 9YA cell lines. p85 is known to recognize and bind the phosphorylated Y608/Y612 residue of mIRS-1/hIRS-1^43^. IGF-1 enhanced such Y608 phosphorylation levels in both wildtype and the mIRS-1 ΔSAR mutants, but this failed to occur in the 9YA mutant which lacks all nine tyrosine residues of the YXXM motifs (Supplementary Fig. S5i). The interactions between mIRS-1 and p85 were significantly elevated upon IGF stimulation (Fig. 5d, lane 1 vs lane 2). In contrast, IGF-1 stimulation had little effect on the interactions between p85 and the mIRS-1 ΔSAR or mIRS-1 9YA mutants (Fig. 5d, lane 2 versus lane 4 or 6). Similarly, neither the ΔSAR or 9YA mutants interacted with endogenous Grb2. We concluded that phase separation of IRS-1 is essential for recruiting downstream p85 and Grb2.

We next determined whether phase separation was required for IRS-1-mediated transduction of insulin/IGF signaling by using wildtype and the engineered C2C12 cell lines. Compared to wildtype cells, the IGF-1-activated AKT and ERK phosphorylation levels were reduced in C2C12-IRS-1 KO myoblasts (Fig. 5e, lane 2 vs lane 4). Only GFP-mIRS-1 (Fig. 5e, lane 4 vs lane 5), but not the ΔSAR or 9YA mutants (Fig. 5e, lane 4 vs lane 6 or 7), rescued the activation of both AKT and ERK. Consistently, the mIRS-1-TDP-43 mutant exhibited a similar stimulatory effect on AKT and ERK activation to the wildtype mIRS-1 (Supplementary Fig. S5j). To further verify whether the droplet formation of IRS-1 represents a promotional event for insulin/IGF signaling or a negative feedback mechanism sequestering the active AKT and ERK kinases, we characterized FoxO1 translocation, another downstream signaling output of insulin/IGF signaling^44^. IGF-1 stimulation resulted in the nuclear export of FoxO1 in C2C12-IRS-1 KO/GFP-mIRS-1 cells, but not in C2C12-IRS-1 KO/GFP-mIRS-1 ΔSAR or C2C12-IRS-1 KO/GFP-mIRS-1 9YA cells (Supplementary Fig. S5k). Given that the phase separation of IRS-1 recruits downstream effectors and is essential for insulin/IGF signaling transduction, we therefore propose that the IRS-1 droplet is an insulin/IGF signalosome.

### Metabolic disease-related IRS-1 G972R mutant displays altered phase transition dynamics

**Fig. 6:**
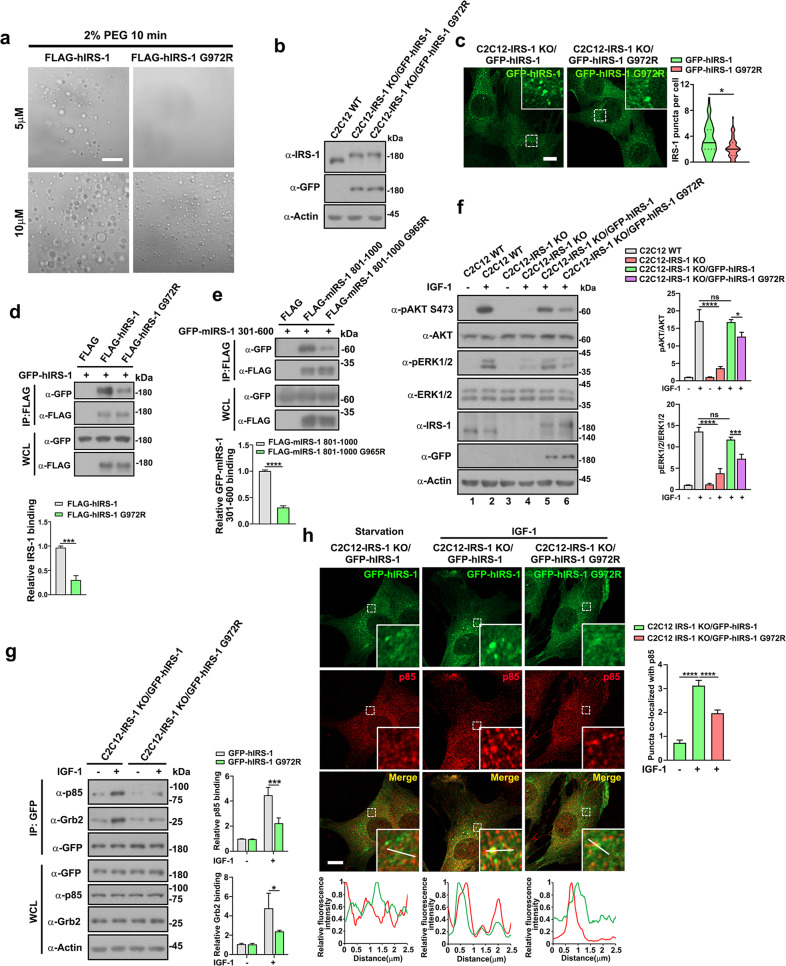
Metabolic disease-related hIRS-1 G972R mutant displays altered phase transition dynamics. **a** DIC images of LLPS of FLAG-hIRS-1 and FLAG-hIRS-1 G972 mutants at a series of protein concentrations (5 or 10 μM). The proteins were incubated with phase separation buffer at room temperature for 10 min. Scale bar, 20 µm**. b** Immunoblot analysis of the IRS-1 expression levels in C2C12 wildtype, C2C12-IRS-1 KO/GFP-hIRS-1, and C2C12-IRS-1 KO/GFP-hIRS-1 G972R cell lines. **c** Confocal images of GFP-tagged hIRS-1 or G972R mutant in C2C12-IRS-1 KO/GFP-hIRS-1 or C2C12-IRS-1 KO/GFP-hIRS-1 G972R cell lines. Scale bar, 10 µm. Quantification results of GFP-hIRS-1 or G972R puncta are shown as violin plots. *p < 0.05. **d** FLAG-tagged hIRS-1 or hIRS-1 G972R mutants were co-transfected with GFP-hIRS-1 into 293 T cells for immunoprecipitation analysis. Data in the bar graphs represent the mean ± SEM values of the ratios of densities for three independent experiments. ***p < 0.001. **e** FLAG-tagged mIRS-1 801–1000 or mIRS-1 801–1000 G965R mutant was co-transfected with GFP-mIRS-1-301-600 into 293 T cells for immunoprecipitation analysis. Data in the bar graphs represent the mean ± SEM values of the ratios of densities for three independent experiments. ****p < 0.0001. **f** Immunoblot analysis of total and phosphorylated AKT and ERK levels in C2C12 wildtype, C2C12-IRS-1 KO, C2C12-IRS-1 KO/GFP-hIRS-1, or C2C12-IRS-1 KO/GFP-hIRS-1 G972R cell lines treated with or without IGF-1 conditional medium for 2.5 min. Data in the bar graphs represent the means ± SEM values of the ratios of densities for three independent experiments. *p < 0.05, ***p < 0.001, ****p < 0.0001. ns: not significant. **g** GFP-tagged hIRS-1 wildtype and G972R mutant were immunoprecipitated in IGF-1-stimulated or control C2C12-IRS-1 KO/GFP-hIRS-1, or C2C12-IRS-1 KO/GFP-hIRS-1 G972R cell lines and then subjected to Western blot with p85 or Grb2 antibodies. Data in the bar graphs represent the mean ± SEM values of the ratios of densities for three independent experiments. *p < 0.05. ***p < 0.001. **h** Confocal images of endogenous p85 and GFP-hIRS-1 or G972R mutants in the indicated cell lines. Scale bar, 10 µm. Line scan showing the related intensity profiles of hIRS-1 with p85. The GFP-hIRS-1 or mutant puncta co-localized with p85 were quantified (*n* = 33). Data in the bar graphs represent the means ± SEM. ****p < 0.0001.

Recent studies have revealed the roles of aberrant phase transition as a mechanism of cellular pathology^45,46^. Polymorphisms in or near the *IRS1* gene are often associated with metabolic diseases in humans^47–50^. In particular, the substitution of the 972 glycine residue for arginine (G972R), which is the most commonly observed polymorphism, has been associated with type 2 diabetes^47,51–53^. Since aberrant LLPS has been established as having links to disease, we considered if the pathogenic G972R mutation impacts the phase separation of IRS-1. To test this hypothesis, we expressed and purified the human IRS-1 wildtype (hIRS-1) and G972R mutant (hIRS-1 G972R) for in vitro phase separation assays (Supplementary Fig. S6a). Notably, hIRS-1 underwent phase separation at a concentration of 5 µM (with 2% PEG) within 10 min, while the metabolic disease-associated mutation could not form foci (Fig. 6a). When the protein concentration was elevated to 10 µM, G972R formed smaller spherical droplets than noted in wildtype hIRS-1 (Fig. 6a), suggesting a reduced ability for undergoing phase separation for the hIRS-1 G972R mutant. We further extended this finding in cells. Using the C2C12-IRS-1 KO cells, we created two myoblast cell lines expressing hIRS-1 (C2C12-IRS-1 KO/GFP-hIRS-1) or hIRS-1 G972R (C2C12-IRS-1 KO/GFP-hIRS-1 G972R) at levels close to those of endogenous IRS-1 (Fig. 6b). Consistent with the in vitro LLPS assays, reduced foci numbers were observed in C2C12-IRS-1 KO/GFP-hIRS-1 G972R cells (Fig. 6c). Ectopically expressed hIRS-1 G972R mutants also consistently displayed a reduced foci volume than in the hIRS-1 wildtype (Supplementary Fig. S6b). Since the self-oligomerization plays essential roles in mediating IRS-1 phase separation, we tested the binding of the G972R mutant to the wildtype hIRS-1. The hIRS-1 G972R mutant did indeed display a weakened association to hIRS-1 (Fig. 6d). The G972 residue localized in the 801–1000 region, which interacts with 301–600 region (Fig. 3e). Consistently, replacing the G965 residue (the corresponding residue of G972 in mIRS-1) to arginine impaired the association of 801–1000 region to 301–600 region (Fig. 6e).

We then further examined the insulin/IGF signaling transduction in the two cell lines. Both cell lines displayed similar tyrosine phosphorylation levels of Y1131/1135/1136 of IGF-1 receptors, and of Y1146/1150/1151 of insulin receptors (Supplementary Fig. S6c). Compared to the C2C12-IRS-1 KO/GFP-hIRS-1 cells, the IGF-1-activated AKT and ERK phosphorylation levels (Fig. 6f), as well as hIRS-1 Y612 phosphorylation levels (Supplementary Fig. S6d), were reduced in C2C12-IRS-1 KO/GFP-hIRS-1 G972R myoblasts. Likewise, the IGF-induced FoxO1 redistribution was attenuated in the C2C12-IRS-1 KO/GFP-hIRS-1 G972R cell line (Supplementary Fig. S6e). We thus considered if the recruitment of p85 or Grb2 to the IRS-1 was hindered due to the impaired phase separation ability of the G972R mutation. Notably, immunoprecipitation analysis revealed a weakened interaction ability of the G972R mutant to endogenous p85 and Grb2 (Fig. 6g). p85 and Grb2 foci consistently colocalized with the IRS-1 droplets in C2C12-IRS-1 KO/GFP-hIRS-1 myoblasts, whereas no such colocalization was identified in C2C12-IRS-1 KO/GFP-hIRS-1 G972R cells (Fig. 6h; Supplementary Fig. S6f). All these findings suggest that aberrant LLPS of IRS-1 is involved in metabolic diseases.

## Discussion

**Fig. 7:**
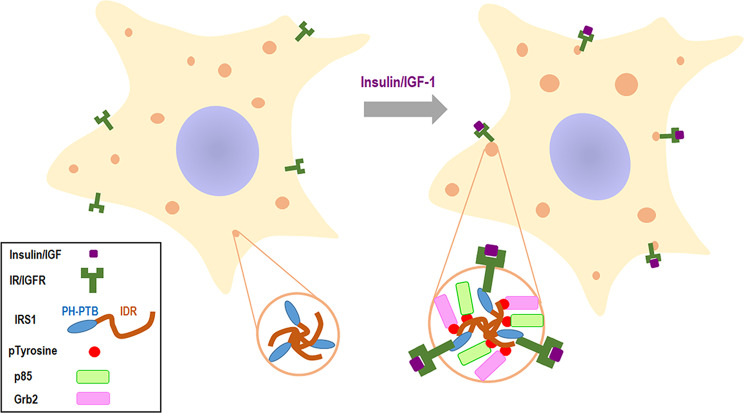
Schematic model for IRS-1-mediated insulin/IGF signalosomes. We found that the C-terminus of IRS-1 undergoes phase separation through mediating self-association. Insulin/IGF signaling leads to tyrosine phosphorylation of IRS-1 which promotes the formation of IRS-1 droplets and the recruitment of downstream effectors to form insulin/IGF signalosomes.

As a pivotal node in insulin/IGF signaling, the activation of IRS-1 needs to be tightly regulated. The IRS-1 carboxyl-terminal tail region is enriched in tyrosine residues that recruit SH2 proteins and in serine/threonine residues that regulate IRS-1 activation^19^. Interestingly, the C-terminus is unstructured with no functional domain. It has been recognized that intrinsically disordered regions (IDRs) of proteins can mediate the inter- or intra-molecular interactions underlying the liquid-like molecular condensations or phase separation^9^. In this study, bioinformatic analysis suggests that the C-terminal tail of IRS-1 might be intrinsically disordered (Fig. 1d; Supplementary Fig. S1d), though this requires validation via more detailed structural analysis. We further demonstrated that the IRS-1 C-terminus mediates the liquid separation of IRS-1 (Fig. 1e; Supplementary Fig. S1f, g). The multivalent interactions of protein-protein or protein-RNA are the major driving forces underlying phase separation-mediated signalosomes^11^. Correspondingly, we identified a key self-association sequence in the carboxyl-terminal tail of IRS-1 that mediates its phase separation (Fig. 3; Supplementary Fig. S3). We also disclosed that insulin/IGF-mediated tyrosine phosphorylation enhanced the self-association of IRS-1 (Fig. 4b; Supplementary Fig. S4b, i). Importantly, deletion of the SAR region, leading to failure of IRS-1 phase separation (Fig. 3), also largely impaired insulin/IGF-evoked AKT and ERK activation (Fig. 5e). These results highlighted self-association-mediated IRS-1 phase separation as a determinant for the insulin/IGF pathway (Fig. 7).

Assembly of dynamic signalosomes is essential for preventing inappropriate signaling caused by inadvertent interactions^12^. One recent study has revealed IDR driving of the LLPS of scaffold protein Axin, which provides a platform for recruiting downstream effectors such as GSK3β and β-catenin^17^. Similarly, the spherical IRS-1 puncta have a highly dynamic liquid-like nature (Fig. 1j, k), and recruit downstream effectors and kinases, including Grb2 and p85 subunit of PI3K, to form insulin/IGF signalosomes (Fig. 5; Supplementary Fig. S5). By doing so they create a high local concentration of insulin/IGF signaling components. Moreover, the membrane-less and liquid-like properties of IRS-1 droplets allow rapid exchange of these signaling molecules between the protein-rich signalosomes and the more diffuse ones in the cytoplasm. This may facilitate both the recruitment of downstream effectors and the transduction of signaling. It has long been recognized that IRS-1 is rapidly phosphorylated upon insulin/IGF stimulation (maximal within 20–40 s)^54^. We thus speculate that the quick response of IRS-1 phosphorylation upon insulin/IGF action may be due to the LLPS of IRS-1.

We recently found that Rab5 interacts with IRS-1 and regulates its activation^31^. In this study we reveal that, even though the IRS-1 sphere lacks a membrane (Fig. 1i), it still associates with Rab5-positive endosomes (Fig. 1a). Inhibition of Rab5 reduced the IRS-1 puncta size and promoted the exchange rate of IRS-1 (Supplementary Fig. S4c, d). This is in line with the recent findings demonstrating intimate interactions between membrane-less condensates and membrane-bound organelles^55^. Indeed, these interactions modulate various functions for both types of organelles. For example, the plasma membrane (PM) supplies platform for the assembly of the condensates of the Nephrin-NCK-N-WASP complex and LAT-ZAP70-GRB2 complex^11,56,57^. Likewise, the ER acts as a location for the biogenesis of phase separations and the modulation of their dynamics^58–60^. A very recent paper pointed out that membranes can reduce the threshold concentration required for condensate formation^60^. Therefore, it remains to be elucidated how Rab5-positive endosomes participate in the biogenesis and dynamics of IRS-1 phase separation.

Aberrant phase separation results in multiple diseases^36,45,61–64^. This includes the disease mutations of FUS, hnRNPA2, Tau, C9orf72 Dipeptide Repeats, and TDP-43, which drive the liquid condensates into more solid-like states^65–70^. It has been well recognized that IRS-1 is crucial for insulin action while the G972 substitution of IRS-1 results in metabolic disorder^47,48,51–53^. Whilst the detailed mechanism remains elusive, both in vitro cell experiments and in vivo transgenic mouse studies have demonstrated the deleterious roles of G972R mutants in insulin action and glucose homeostasis^71–73^. Here, we found that the metabolic disease-associated G972R mutation undermined the ability of IRS-1 to undergo phase separation in vitro (Fig. 6a) and in cells (Fig. 6c; Supplementary Fig. S6b). In line with these findings, G972R polymorphism impairs the self-association of IRS-1 (Fig. 6d) that is essential for mediating phase separation (Fig. 5). We thus propose that the pathological effects of the G972R mutation might be due to the impairment of IRS-1-mediated insulin/IGF signalosomes. These results thus strongly implicate aberrant IRS-1 phase separation in metabolic diseases. Recent studies have revealed the involvement of phase separation in modulating pivotal cellular metabolic events including lipid droplet formation and autophagy^74,75^. All such findings may provide new opportunities for therapeutic interventions in cases of metabolic disease.

Post-translational modifications (PTMs) can modulate protein interaction strength to influence phase separation^76,77^. As a reversible and fast PTM, phosphorylation can quickly respond to different cues to regulate biomolecular condensation. For example, the phosphorylation of Tau promotes its phase separation by enhancing electrostatic interactions^78^. Likewise, phase separation of PGL-1/-3 and Mxc were accelerated by mTOR-mediated and CDK-mediated phosphorylation, respectively^79,80^. Multiple tyrosine residues of LAT and nephrin can also be phosphorylated to form multivalent condensates by recruiting Grb2 and Nck^14,56,57^. We also found that replacing the tyrosine residues in the intrinsically disordered region of IRS-1 inhibits its phase separation (Fig. 4). This disclosed the importance of tyrosine phosphorylation in modulating the multivalent interactions of IRS-1. Other PTMs including glycosylation, acetylation, methylation, and PARylation have also been found to promote or suppress biomolecule condensates in context-dependent manners^81–88^. It is thus of interest to further investigate if other types of PTMs provide additional levels of control on IRS-1 mediated signalosomes.

## Materials and methods

### Culture and maintenance of cells

The C2C12 and MCF7 cell lines were from Cell bank of the Chinese Academy of Sciences. The HEK293T cell line was from American Type Culture Collection. C2C12 cells and MCF7 cells were grown in DMEM (high glucose) supplemented with 15% (v/v) or 10% fetal bovine serum respectively. 293T cells were grown in RPMI-1640 medium supplemented with 10% (v/v) fetal bovine serum, 2mM L-glutamine, 100 U/mL penicillin and 100 mg/mL streptomycin (all from Hyclone Laboratories, Logan, UT). Cells in six-well plates were transfected with lipofectamine 2000 (Invitrogen) according to the manufacturer’s protocol.

### Antibodies

The following antibodies were purchased from Cell Signaling Technology: rabbit anti-IRS-1 (2382), rabbit anti-pAKT S473 (9271), rabbit anti-p85 (4292), rabbit anti-Grb2 (3972), rabbit anti-FoxO1 (2880), rabbit anti-ERK1/2 (9102), rabbit anti-pERK1/2 (4370), anti-IGF-1Rβ (9750), anti-IRβ (3025), anti-pIGF-1Rβ Y1135/1136/pIRβ Y1150/1151 (3024), anti-pIGF-1Rβ Y1131/pIRβ 1146 (3021). Other antibodies were from the following commercial sources: rabbit anti-pIRS-1 Y612 (Invitrogen, 44-816 G); rabbit anti-AKT (HUABIO, ET1609-47), rabbit anti-GFP (HUABIO, ET1607-31), mouse anti-Actin (HUABIO, M1210-2), mouse anti-MHC (DSHB, MF20), mouse anti-FLAG (YEASEN, 30503ES60), and mouse anti-mCherry (ABclonal, AE002). HRP-conjugated secondary antibodies: goat anti-mouse second antibody (Jackson, 115-035-003) and goat anti-rabbit second antibody (Jackson, 111-035-003). Fluorescent secondary antibodies were from Thermo Fisher Scientific: goat anti-mouse-Alexa Fluor 546 (A-11003), goat anti-rabbit-Alexa Fluor 488 (A-11008), goat anti-rabbit-Alexa Fluor 546 (A-11010), goat anti-rabbit-Alexa Fluor 633 (A-21071).

### Plasmids

Full-length cDNAs of mIRS-1, mIRS-2, hIRS-1, p85, Grb2 and mutant proteins were cloned into a hemagglutinin (HA)-tagged, GFP-tagged, and FLAG-tagged pXJ40 expression vector (E Manser, IMCB, Singapore). All plasmids were purified using an Axygen miniprep kit for use in transfection experiments. *Escherichia coli* strain DH5-α was used as a host for propagation of the clone. All the mutations used in this study were created using the standard PCR-based mutagenesis method and confirmed by DNA sequencing. The plasmid information was provided in Supplementary Table S1.

### IGF-1/insulin stimulation

C2C12 cells or HEK293T cells were serum-starved for 12 hours in DMEM, and then treated with 100 ng/mL IGF-1 (Sino biological, 10598-HNAY1) or 100 nM insulin (Sigma, I0305000) for the indicated times.

### Protein expression and purification

The plasmids of FLAG-tagged mIRS-1, hIRS-1 or their mutants were transfected into 293 T cells for 2 days. The Cells were harvested by centrifugation at 14,000 rpm for 10 min at 4 °C and lysed with HEPES buffer (150 mM sodium chloride, 50 mM HEPES, pH 7.4, 5 mM EDTA, 1% (w/v) sodium deoxycholate, 1% (v/v) Triton X-100, 0.2% sodium fluoride, 0.1% sodium orthovanadate, and protease Inhibitor cocktail (Selleck Chemicals, B14001)). Anti-FLAG M2 gel beads (Bimake, B23102) were then added and incubated on a rotary shaker at 4 °C for 2 h. M2 gel beads were harvested by centrifugation at 3,000 rpm for 1 min and washed four times in HEPES buffer. The FLAG-tagged proteins were purified by the competition of 3× FLAG peptide (MCE, HY-P0319). Briefly, M2 beads were resuspended with 1.5 mg/mL 3× FLAG peptide buffer and incubated at 4 °C for 2 h. After centrifugation at 5,000 rpm for 1 min, the supernatant further purified by gel filtration using a Superdex^TM^ 200 increase column (GE Healthcare, 28-9909-44) equilibrated in store buffer (50 mM Tris-HCl, 37 mM NaCI, 1 mM EDTA, 5 mM DTT). All the purified proteins were concentrated by centrifugal filtration (Millipore) and stored in aliquots at –80 °C. The purified protein was quantified using a ND-2000 NanoDrop spectrophotometer (Thermo scientific) with OD 280 and verified by Coomassie staining.

### CRISPR/Cas9 knock-out in C2C12 cells

The CRISPR/Cas9-based IRS-1 knockout C2C12 cell line was generated as previously reported^36^. Oligos coding for guide RNAs targeting the N terminus of IRS-1 were cloned into a lentiCRISPRv2 backbone. The sequence targeted for IRS-1 was 5′-GCATACTCTTGGGCTTGCGC-3′. Cloned plasmids were transfected by Lipofectamine 3000 (Thermo Fisher Scientific, L3000015). After 24 h, cells were selected using 5 μg/mL puromycin (GPC Biotechnology, AK058). After two days of selection, cells were sorted into single cells by flow cytometric cell sorting and seeded into 96-well plates. IRS-1 KO single-cell clones were then screened by Western blot assay.

### Generation of inducible stable cell line

A tet-on system was used for C2C12 cells or the IRS-1 KO cell line to generate inducible stable cell lines as previously described^89^. Cells were co-transfected with HP216 vector and HP138-GFP-IRS-1-related vectors. After 24 h, cells were treated with 500 ng/mL doxycycline (YEASEN, 60204ES03) for 1 day to induce the expression of proteins and sorted by flow cytometric fluorescence sorting (Beckman moflo Astrios EQ). Western blot was performed to validate the expression levels.

### Estimation of endogenous IRS-1 protein concentrations

The concentration of endogenous IRS-1 protein was measured following the previously reported protocol^36,90^. Briefly, quantification was based on the western blot densitometry analysis performed on cell lysates and purified FLAG-mIRS-1 or FLAG-hIRS-1 protein. C2C12 cells or HEK293T cells were lysed in WB lysis buffer with protease inhibitors and subjected to western blot with purified FLAG-mIRS-1 or FLAG-hIRS-1 protein. After densitometry analysis of western blot result using Fiji, we plotted band density against the purified FLAG-mIRS-1 or FLAG-hIRS-1 concentrations.

### In vitro phase separation assays

The purified proteins were added to phase separation Buffer (50 mM Tris-HCl pH 7.5, 10% glycerol, 1 mM DTT, 2% PEG-8000 (Sangon biotech, A100159)). The concentration of NaCl was adjusted to the indicated concentrations. The protein solution was loaded onto an 8-well chamber (Cellvis, C8-1.5H-N) for 5 min at room temperature and then imaged using a Zeiss LSM 800 confocal microscope with a 63× objective (Carl Zeiss). For temperature-mediated phase separation, the 8-well chamber was first incubated in 37 °C for 5 min and then shifted to 4 °C for 5 min. For 1,6-Hexanediol (TCI, H0099)-mediated phase separation, 1,6-Hexanediol was present at the indicated concentrations. The concentrated proteins were treated with 0.1 mg/mL RNase A (Axygen, AP-MD-P), 1 U DNase (Thermo Scientific, EN0521) or 0.1 mg/mL BSA for 1 h at room temperature to examine the effects of RNA or DNA on LLPS. Droplet turbidity OD600 was measured by using a Thermo Multiskan Sky microplate reader.

### Protein fluorescence labeling

iFluor^TM^ 488 NHS ester (AAT Bioquest, 1023) were dissolved in DMSO and incubated with IRS-1 protein at room temperature for 1 h (fluorophore to protein molar ratio was 1:1). The fluorophores and other small molecules were removed from the proteins by using a Microcon-100-kDa Centrifugal Filter Unit with Ultra-100 membrane (Millipore, UFC810024) with store buffer (50 mM Tris-HCl, 37 mM NaCI, 1 mM EDTA, 5 mM DTT).

### Generation of phase diagram

Phase diagrams were generated by mixing mIRS-1 or its mutant protein at concentrations varying from 0.1 to 1.6 μM, in a phase separation buffer with sodium chloride concentrations varying from 50–1200 mM. Droplet turbidity OD600 was then measured. For turbidity measurements, samples were incubated at 37 °C for 2 h and 15 μL of each sample was added into 384-well white polystyrene plate with clear flat bottom, and the value of OD600 was measured using a microplate reader as described above.

### Fluorescence recovery after photobleaching (FRAP)

FRAP experiments were performed on a Zeiss LSM 800 microscope with a 63× oil immersion objective. C2C12 cells were seeded onto 8-well chamber slides (Cellvis, C8-1.5H-N). Cells were transfected with GFP-IRS-1 plasmids. After 24 h incubation, GFP-IRS-1 droplets were photobleached using a laser intensity of 80% at 480 nm (for GFP) and recovery was recorded for the indicated time. iFluor^TM^ 488-FLAG-mIRS-1 droplets were photobleached using a laser intensity of 100% at 480 nm. The prebleached fluorescence intensity was normalized to 1 and the signal after bleaching was normalized to the pre-bleached level.

### Immunoprecipitation studies and Western blot analyses

Control cells or cells transfected with expression plasmids were lysed in lysis buffer (150 mM sodium chloride, 50 mM Tris, pH 7.3, 0.25 mM EDTA, 1% (w/v) sodium deoxycholate, 1% (v/v) Triton X-100, 0.2% sodium fluoride, 0.1% sodium orthovanadate, and protease Inhibitor cocktail (Selleck Chemicals, B14001). Lysates were immunoprecipitated (IP) with anti-FLAG M2 beads (Bimake, B23102). Samples were run in SDS/PAGE gels and analyzed by Western blotting with the indicated antibodies.

### Immunofluorescence and direct fluorescence studies

Cells were seeded on coverslips in 24-well plates and transfected with various expression constructs for 24–36 h. They were then stained for immunofluorescence detection using confocal fluorescence microscopy or directly visualized for cells expressing GFP or mCherry-tagged proteins as previously described^91^. Briefly, the cells were washed with PBSCM buffer (PBS buffer supplemented with 10 mM CaCl_2_ and 10 mM MgCl_2_) followed by fixation with 3% paraformaldehyde in PBSCM. The fixed cells were then washed with PBSCM containing 50 mM NH_4_Cl and permeabilized with PBSCM containing 0.1% saponin. For immunostaining, the antibodies were diluted in PBSCM containing 7% fetal bovine serum and 2% bovine serum albumin. Images were collected using a 63× oil immersion objective with appropriate laser excitation on a Zeiss LSM 800 confocal microscope. The detector gain was first optimized by sampling various regions of the coverslip and then fixed for each specified channel. Once set, the detector gain value was kept constant throughout the image acquisition process. Images were analyzed using Zeiss LSM Image Examiner Software.

### Puncta size measurement

The GFP-mIRS-1 droplets that colocalize with the Ab-IRS-1-pY608, p85 or Grb2 were calculated by Fiji. Briefly, the colocalization regions with scale bar were chosen as ROI and the ROI scale was firstly set in Fiji. The straight line was drawn and measured in droplets.

### Domain and disorder prediction

The intrinsically disordered region of mouse IRS-1, human IRS-1, or human IRS-2 was identified with the use of PONDR (http://www.pondr.com/).

### Three-dimensional (3D) rendering, sphericity, volume and number measurement

C2C12 cells transiently transfected with GFP-IRS-1 were fixed with 3% paraformaldehyde. Z stack images were acquired using a Zeiss LSM 800 confocal microscope. The step size was 0.12 µm. 3D rendering was performed using Imaris software. Sphericity, volume, and number were also calculated using Imaris software.

### Correlative confocal and electron microscopy

C2C12 cells were plated on glass gridded coverslips (Cellvis, D35-14-1.5GI) and transfected with indicated plasmids. 24 h after transfection, cells were fixed with 3% paraformaldehyde for 20 min and imaged on Zeiss Airyscan to collect light microscopy images. The cells were then fixed with 2.5% glutaraldehyde for 12 h at 4 °C and postfixed in 2% osmium tetroxide-3% potassium ferrocyanide in cacodylate buffer for 1 h followed by 1% thiocarbohydrazide dissolved in water for 20 min and incubated in 2% osmium in cacodylate buffer for 30 min. Samples were then dehydrated with a graded ethanol series (20%, 50%, 70%, 90%, and 100%) for 15 min each and processed for Epon embedding. The samples were cut (30 KV and 2.5 nA) and imaged (2 KV and 0.2 nA) by FIB-SEM (Helios UC G3).

### Statistical analysis

Statistical analyses were performed in GraphPad Prism 8.0.2 (GraphPad Software, Inc.). Results are presented as mean ± SEM or means ± SD. Statistical significance was determined as indicated in the figure legends: *p < 0.05, **p < 0.01, ***p < 0.001, ****p < 0.0001. The data distribution was first checked using a Shapiro-Wilk normality test, Kolmogorov–Smirnov test and D’Agostino & Pearson omnibus normality test. For comparison between two groups and if the data fitted a normal distribution, a two-tailed unpaired Student’s *t*-test was used when variances were confirmed as similar via an F test (*p* > 0.05). A two-tailed unpaired Student’s *t*-test with Welch’s correction was used when variances were shown up as different via the F test (*p* < 0.05). If the data did not fit a normal distribution, a Mann-Whitney test was used. If the variation among three or more groups was minimal, ANOVA followed by Dunnett’s post-test or Tukey’s post hoc test was applied for comparison of multiple groups.

## Supplementary information

Supplementary information, Figures and Table

## Data Availability

The source data from this study are provided along with the paper. Raw image files are available upon request.
