# Supplementary material for: Phase separation of insulin receptor substrate 1 drives the formation of insulin/IGF-1 signalosomes
Source: Cell Discov. 2022 Jun 28;8:60. doi: 10.1038/s41421-022-00426-x (PMC9240053; doi:10.1038/s41421-022-00426-x)
Supplement: Supplementary file 1 — Supplementary information, Figures and Table [file 41421_2022_426_MOESM1_ESM.pdf]

Supplemental Figures and Legends

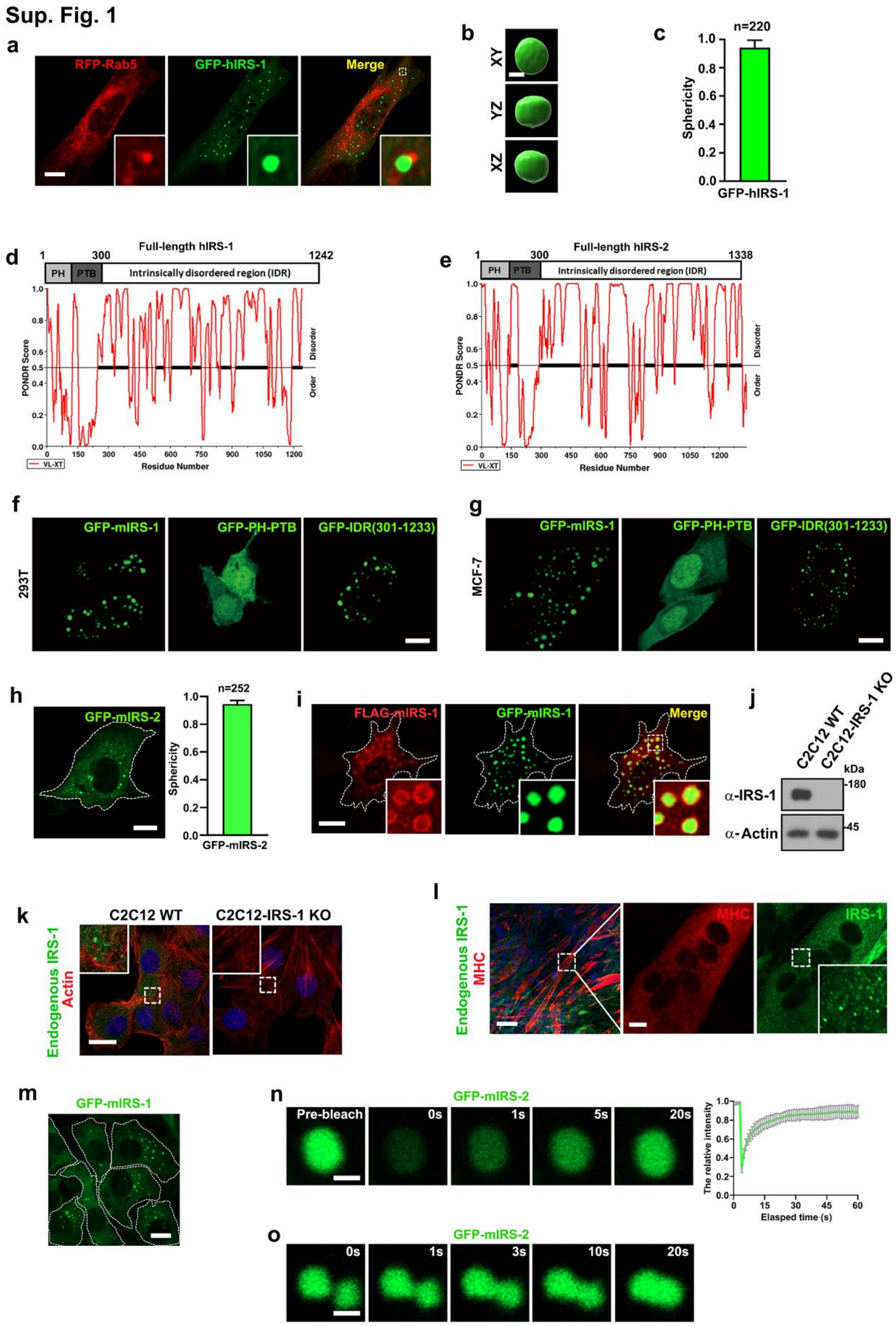

Fig S1. Phase separation of IRS-1 mediated by its C-terminus. a Confocal images of representative C2C12 myoblasts co-expressing RFP-Rab5 and GFP-hIRS-1. Scale

bar, 10 µm. **b** Rendered 3D shapes of an hIRS-1 droplet. The panels show the XY, XZ, and YZ planes. Scale bar, 1 µm. **c** A plot showing the sphericity of hIRS-1 droplets (n=220). Data are shown as mean ± SEM. **d** Protein sequence and disorder prediction (PONDR) of the hIRS-1 [1242 amino acids (aa)]. **e** Protein sequence and disorder prediction (PONDR) of the hIRS-2 [1338 amino acids (aa)]. **f** Representative images of mIRS-1 droplets in 293T cells expressing either GFP-tagged mIRS-1, the PH-PTB region (1-300 amino acids), or mIRS-1 IDR (301-1233 amino acids). Scale bar, 10 µm. **g** Representative images of mIRS-1 droplets in MCF-7 cells expressing either GFP-tagged mIRS-1, the PH-PTB region (1-300 amino acids), or mIRS-1 IDR (301-1233 amino acids). Scale bar, 10 µm. **h** Representative images of GFP-mIRS-2 droplets in C2C12 cells. Scale bar, 10 µm. A plot showing the sphericity of GFP-mIRS-2 droplets (n=252). Data are shown as mean ± SEM. **i** Representative confocal images of C2C12 myoblasts co-expressing GFP-tagged mIRS-1 and FLAG-tagged-mIRS-1. Scale bar, 10 µm. **j** Immunoblot analysis of endogenous IRS-1 expression levels in C2C12 wildtype and C2C12-IRS-1 KO cell lines. **k** Confocal images of endogenous IRS-1 in C2C12 wildtype and C2C12-IRS-1 KO cell lines. Scale bar, 20 µm. **l** Confocal images of endogenous IRS-1 in differentiated C2C12 myotubes. Scale bar, 100 µm. the inset scale bar, 10 µm. **m** Similar expression levels of GFP-mIRS-1 in DOX-induced C2C12-IRS-1 KO/GFP-mIRS-1 cells. Scale bar, 10 µm. **n** Confocal images and quantification of GFP-mIRS-2 fluorescence recovery after photobleaching (n=14). Scale bar, 1 µm. **o** Time-lapse imaging showing fusion of two GFP-mIRS-2 droplets in cells. Scale bar, 1 µm.

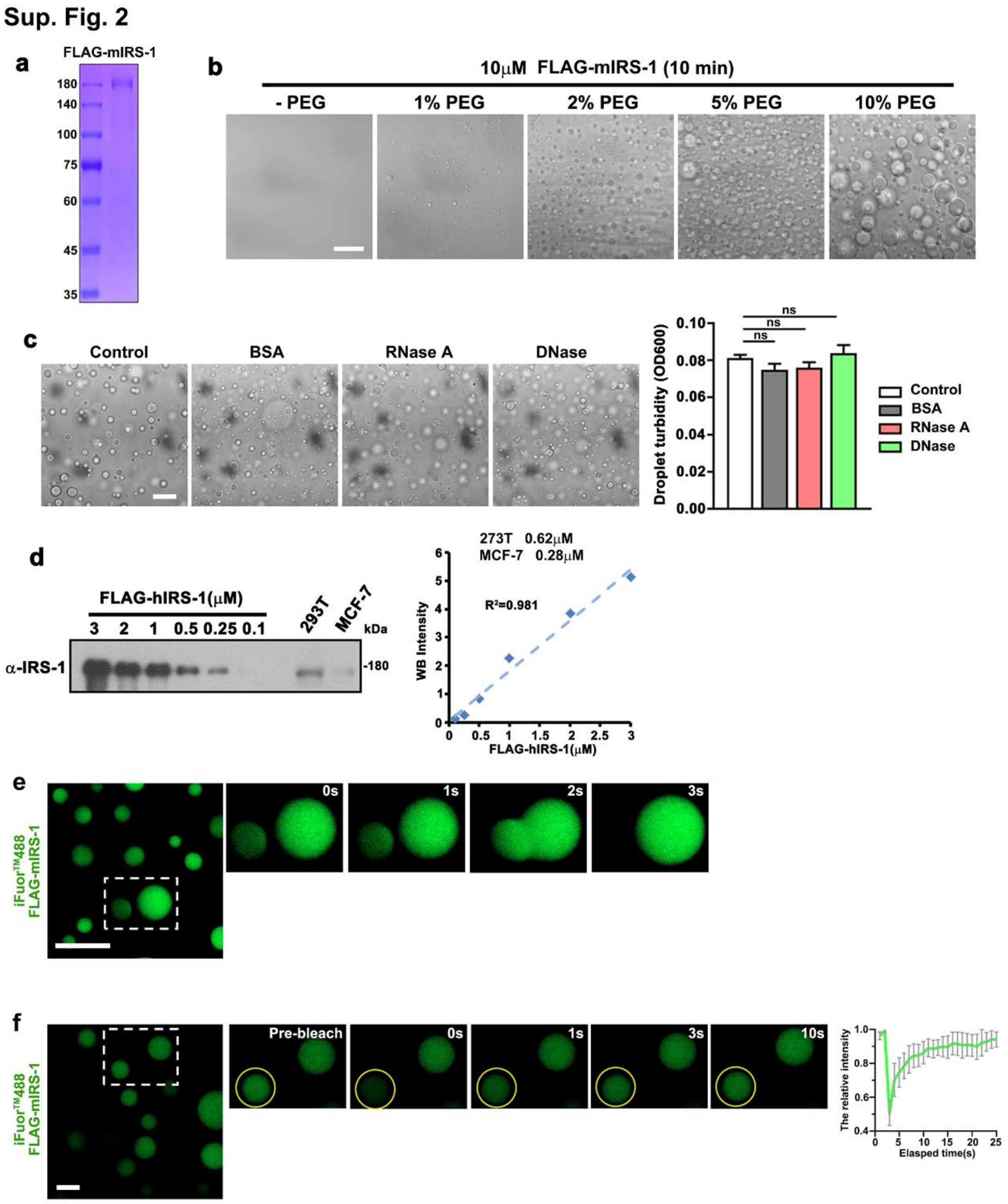

Fig S2. Crowding reagent enhances the phase separation of IRS-1. a SDS-PAGE and Coomassie blue staining results of purified recombinant FLAG-mIRS-1 protein. **b** DIC images of FLAG-mIRS-1 (10μM) LLPS at a series of PEG-8000 concentration (1-10%). The proteins were incubated with phase separation buffer at room temperature for 10 min. **c** The proteins (1μM) were treated with indicated reagents for 1 hour at

room temperature. The quantification result is shown as mean ± SD. ns: not significant. **d** Quantification result of endogenous IRS-1 protein concentration in 293T and MCF7 cells based on immunoblot densitometry analysis performed on cell lysates and purified FLAG-hIRS-1 protein. **e** Time-lapse imaging showing fusion of two iFluor™ 488-FLAG-mIRS-1 droplets in buffer at room temperature. Scale bar, 10 μm. **f** Confocal images and quantification of iFluor™ 488-FLAG-mIRS-1 fluorescence recovery after photobleaching (n=11). Data are shown as mean ± SD. Scale bar, 10 μm.

Sup. Fig. 3

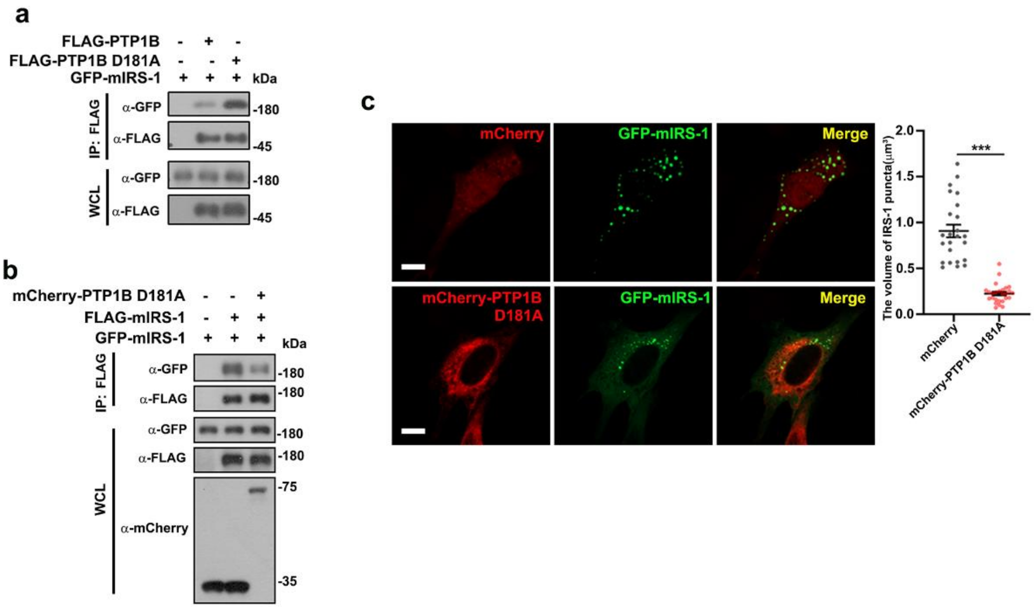

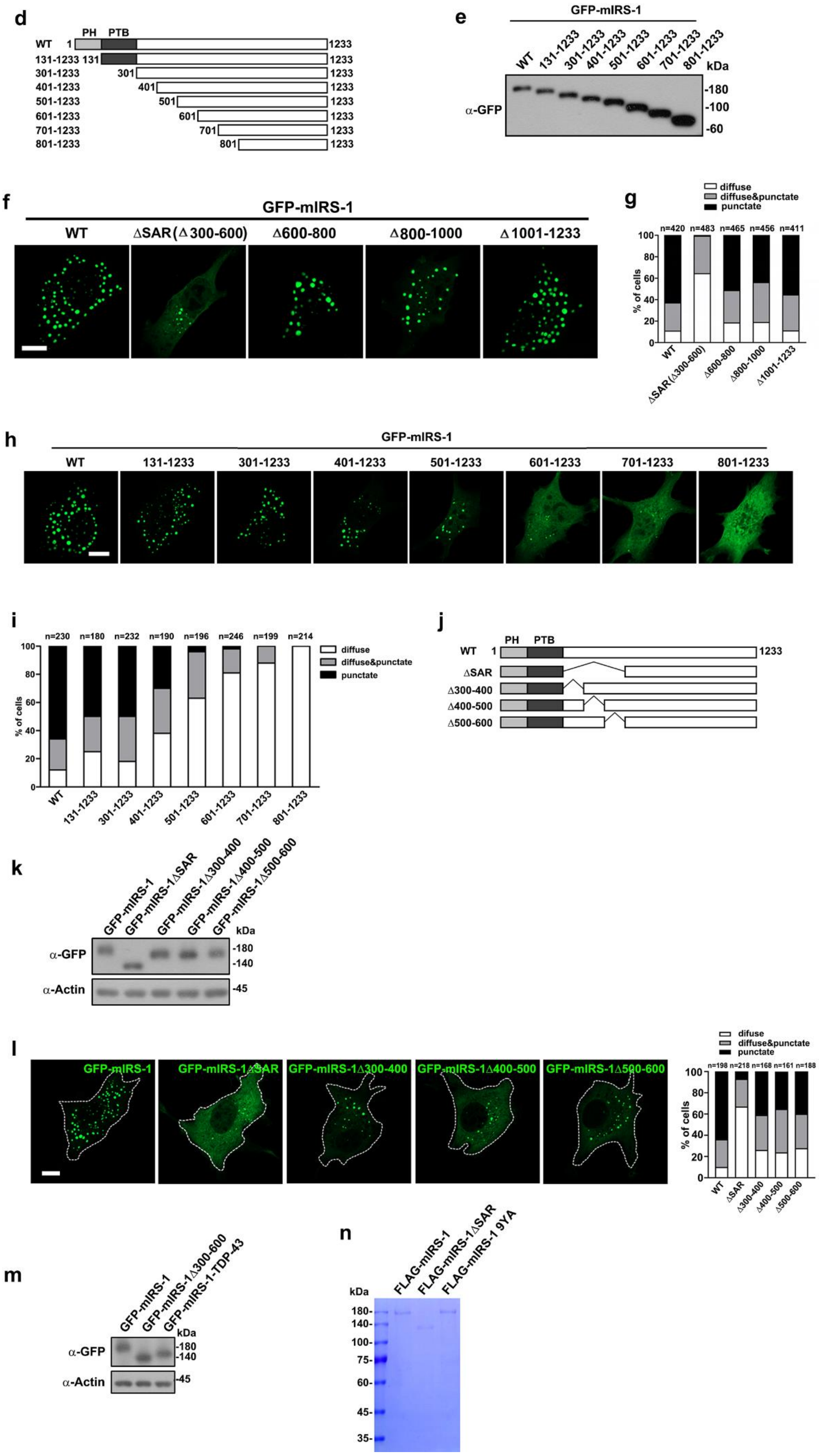
d
PH PTB
WT 1 1233
131-1233 131 1233
301-1233 301 1233
401-1233 401 1233
501-1233 501 1233
601-1233 601 1233
701-1233 701 1233
801-1233 801 1233
e
GFP-mIRS-1
WT 131-1233 301-1233 401-1233 501-1233 601-1233 701-1233 801-1233
kDa
α-GFP
-180
-100
-60
f
GFP-mIRS-1
WT ΔSAR(Δ300-600) Δ600-800 Δ800-1000 Δ1001-1233
g
diffuse
diffuse&punctate
punctate
n=420 n=483 n=465 n=456 n=411
% of cells
WT ΔSAR(Δ300-600) Δ600-800 Δ800-1000 Δ1001-1233
h
GFP-mIRS-1
WT 131-1233 301-1233 401-1233 501-1233 601-1233 701-1233 801-1233
i
n=230 n=180 n=232 n=190 n=196 n=246 n=199 n=214
diffuse
diffuse&punctate
punctate
% of cells
WT 131-1233 301-1233 401-1233 501-1233 601-1233 701-1233 801-1233
j
PH PTB
WT 1 1233
ΔSAR
Δ300-400
Δ400-500
Δ500-600
k
GFP-mIRS-1 GFP-mIRS-1ΔSAR GFP-mIRS-1Δ300-400 GFP-mIRS-1Δ400-500 GFP-mIRS-1Δ500-600
kDa
α-GFP -180 -140
α-Actin -45
l
GFP-mIRS-1 GFP-mIRS-1ΔSAR GFP-mIRS-1Δ300-400 GFP-mIRS-1Δ400-500 GFP-mIRS-1Δ500-600
difuse
diffuse&punctate
punctate
n=198 n=218 n=168 n=161 n=188
% of cells
WT ΔSAR Δ300-400 Δ400-500 Δ500-600
m
GFP-mIRS-1 GFP-mIRS-1Δ300-600 GFP-mIRS-1-TDP-43
kDa
α-GFP -180 -140
α-Actin -45
n
FLAG-mIRS-1 FLAG-mIRS-1ΔSAR FLAG-mIRS-1 9YA
kDa
180- 140- 100- 75- 60- 45- 35-

Fig S3. The 300-600 region of mIRS-1 is essential for phase separation.

a FLAG-tagged PTP1B wildtype or D181A mutant construct was co-transfected with GFP-mIRS-1 into 293T cells for immunoprecipitation analysis. **b** mCherry-tagged PTP1B D181A mutant was co-transfected with FLAG-tagged and GFP-tagged mIRS-1 into 293T cells for immunoprecipitation analysis. **c** Confocal images of representative C2C12 myoblasts co-expressing GFP-mIRS-1 and mCherry vector or mCherry-PTP1B D181A mutant. The volume of GFP-mIRS-1 puncta was quantified (n=26). Data in the graphs represent the mean ± SEM. ***: $p<0.001$. Scale bar, 10 μm. **d** Schematic diagram of mIRS-1 and its truncation mutants. **e** GFP-tagged mIRS-1 mutant constructs as shown in **d** were expressed in 293T cells. **f** Confocal images of representative C2C12 myoblasts expressing GFP-tagged mIRS-1 mutants as shown in **Fig. 3c**. Scale bar, 10 μm. **g** Quantitative analysis of phase separation of GFP-tagged mIRS-1 mutants as shown in **f** (classed as predominantly diffuse, diffuse plus punctate, or predominantly punctate). **h** Confocal images of representative C2C12 myoblasts expressing GFP-tagged mIRS-1 mutants as shown in **d**. Scale bar, 10 μm. **i** Quantitative analysis of phase separation of GFP-tagged mIRS-1 mutants as shown in **h** (classed as predominantly diffuse, diffuse plus punctate, or predominantly punctate). **j** Schematic diagram of mIRS-1 and its deletion mutants. **k** Immunoblot analysis of expression levels of the indicated GFP-tagged mutant proteins as shown in **j**. **l** Confocal images of representative C2C12 myoblasts expressing GFP-tagged mIRS-1 mutants as shown in **j**. Scale bar, 10 μm. **m** Immunoblot analysis of GFP-tagged mIRS-1 and mutants expression levels in Fig. 3f. **n** SDS-PAGE and Coomassie blue staining results of

purified recombinant FLAG-mIRS-1 wildtype and mutant proteins.

Sup. Fig. 4

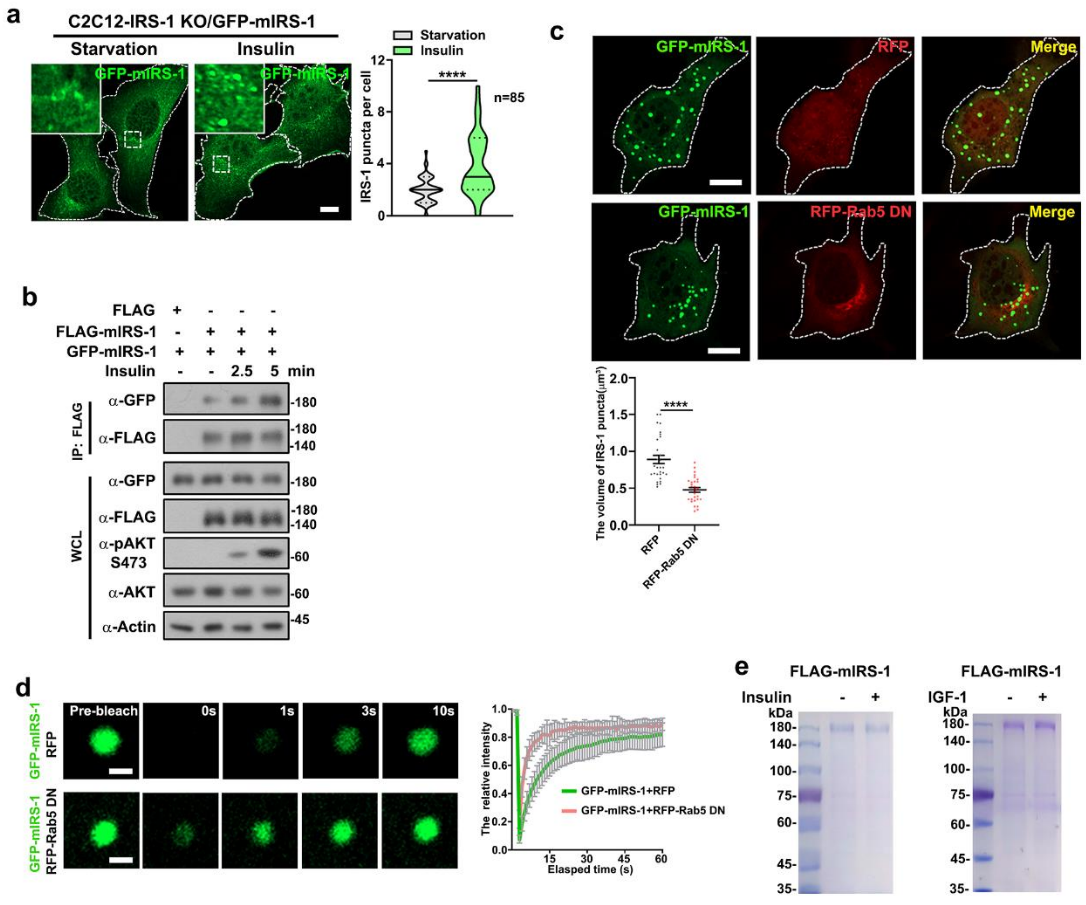

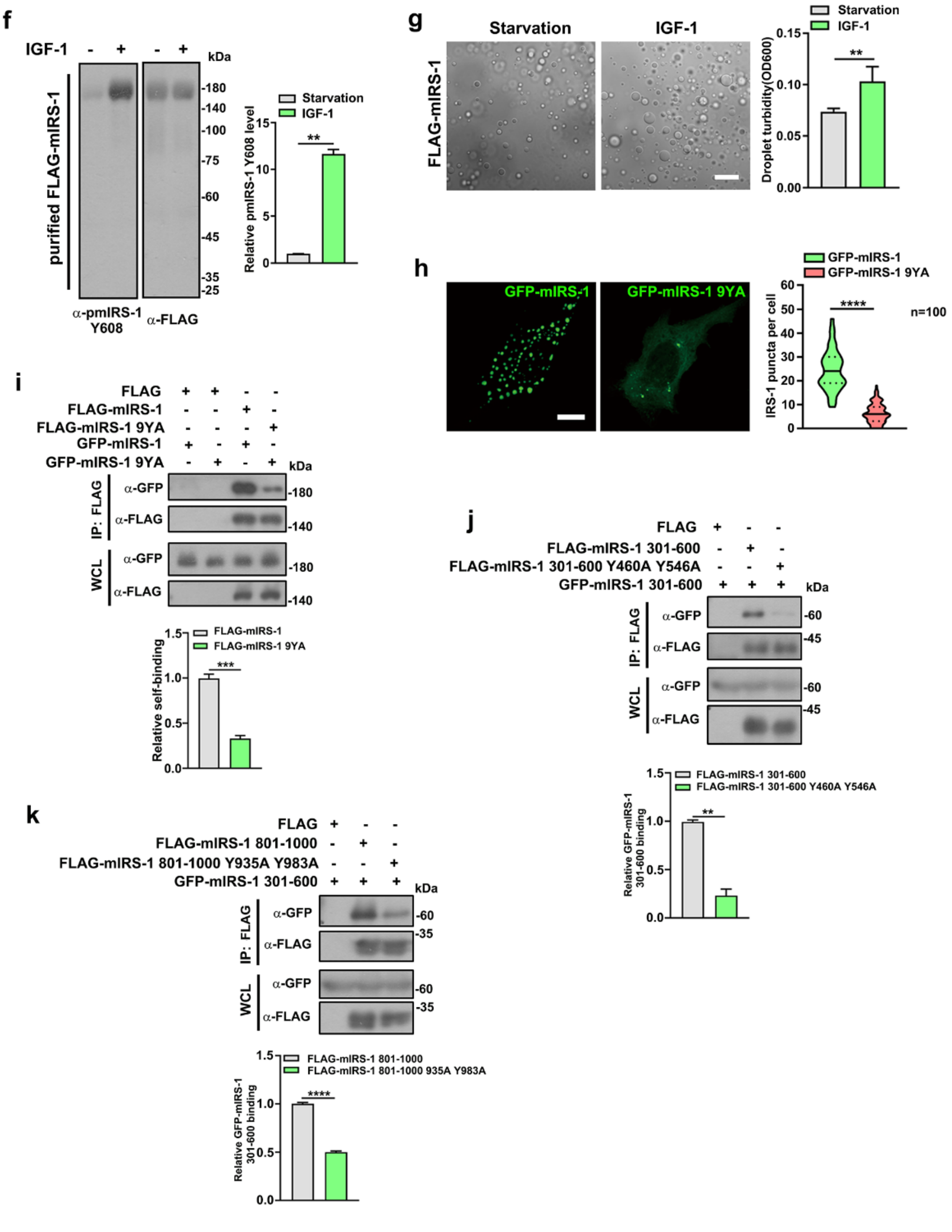

Fig. S4 Insulin/IGF-1 stimulation promotes the phase separation of IRS-1.

a Confocal image of GFP-mIRS-1 foci in C2C12-IRS-1 KO/GFP-mIRS-1 cells treated with control or with insulin-conditioned (100nM) medium for 2.5 min. Scale bar, 10 μm. Quantitative analysis of the number of mIRS-1 puncta with data shown as a violin plot. ****: p<0.0001. **b** FLAG-tagged and GFP-tagged mIRS-1 were co-transfected into 293T cells. Cells were serum starved for 16 hours followed by insulin stimulation

and coimmunoprecipitation analysis. **c** Confocal images of representative C2C12 myoblasts co-expressing GFP-mIRS-1 and RFP vector or RFP-Rab5 DN mutant. Scale bar, 10 μm. The puncta volume was quantified (n=30). ****: $p<0.0001$. **d** Confocal images and quantification of GFP-mIRS-1 fluorescence recovery after photobleaching (n=15). Data are shown as mean ± SD. Scale bar, 1 μm. **e** SDS-PAGE and Coomassie blue staining results of FLAG-mIRS-1 proteins purified from insulin- or IGF-1-stimulated cells. **f** Immunoblot analysis of Y608 tyrosine phosphorylation of FLAG-mIRS-1 purified from starved or IGF-1-stimulated (15min) 293T cells. The quantification result is shown as mean ± SEM. **: $p<0.01$. **g** DIC images of FLAG-mIRS-1 purified from starved or IGF-1-stimulated (15min) 293T cells. The proteins (1μM) were incubated with phase separation buffer at room temperature for 20 min. Scale bar, 20 μm. The quantification result is shown as mean ± SD. **: $p<0.01$. **h** Confocal images of representative C2C12 cells expressing either GFP-tagged mIRS-1 or GFP-mIRS-1 9YA mutant. Scale bar, 10 μm. The quantification result is also shown as violin plot. ****: $p<0.0001$. **i** FLAG-tagged and GFP-tagged mIRS-1 or mIRS-1 9YA mutant were co-transfected into 293T cells for immunoprecipitation analysis. **j** FLAG-tagged 301-600 or 301-600 Y460A Y546A mutant were co-transfected with GFP-tagged 301-600 into 293T cells for immunoprecipitation analysis. **k** FLAG-tagged 801-1000 or 801-1000 Y935A Y983A mutant were co-transfected with GFP-301-600 into 293T cells for immunoprecipitation analysis. Data in the bar graphs represent the mean ± SEM values of the ratios of densities for three independent experiments in i, j and k. **: $p<0.01$. ***: $p<0.001$. ****: $p<0.0001$.

Sup. Fig. 5

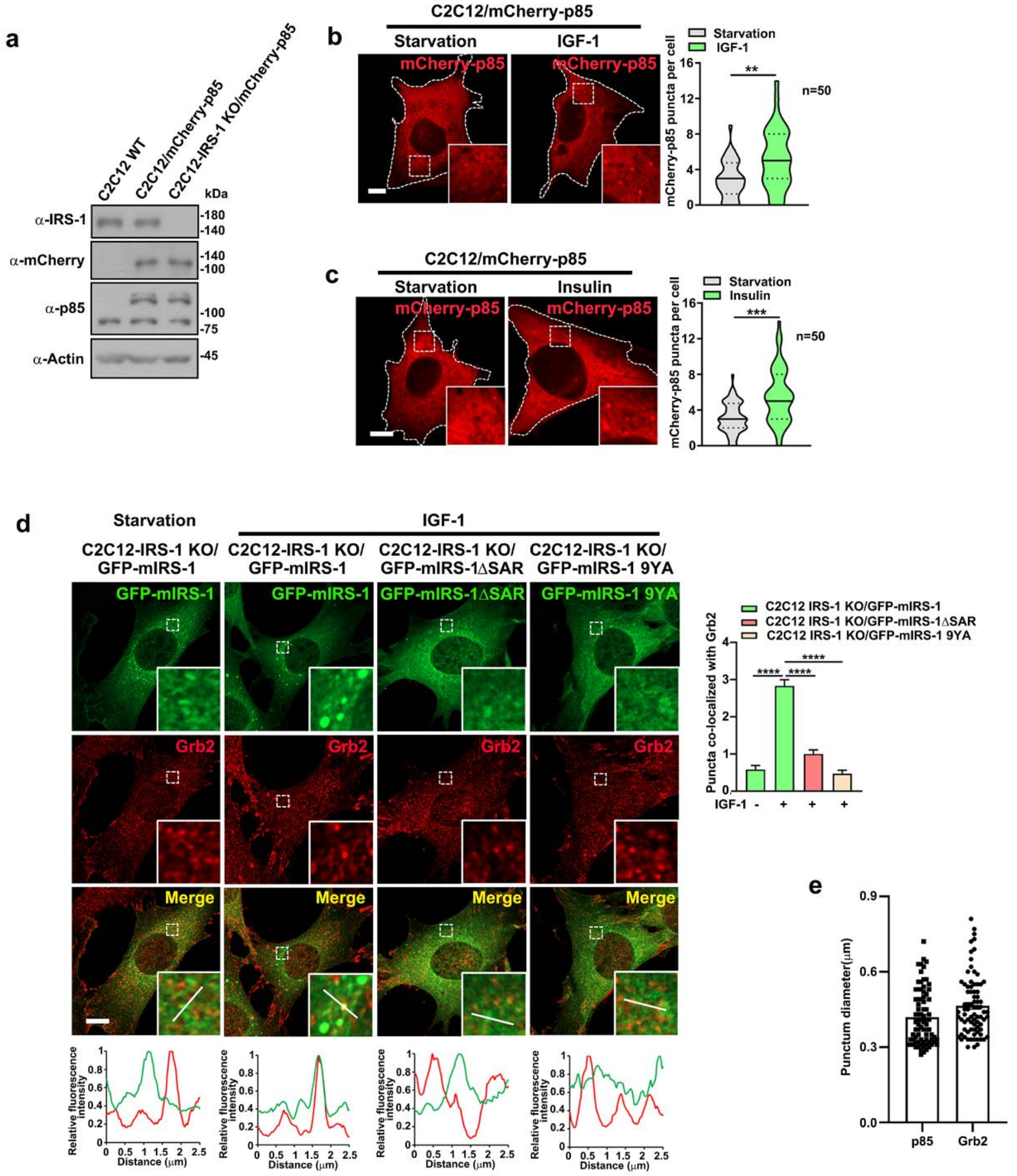
a
C2C12 WT
C2C12/mCherry-p85
C2C12-IRS-1 KO/mCherry-p85
kDa
α-IRS-1
α-mCherry
α-p85
α-Actin
-180
-140
-140
-100
-100
-75
-45
b
C2C12/mCherry-p85
Starvation
IGF-1
mCherry-p85
Starvation
IGF-1
mCherry-p85 puncta per cell
n=50
c
C2C12/mCherry-p85
Starvation
Insulin
mCherry-p85
Starvation
Insulin
mCherry-p85 puncta per cell
n=50
d
Starvation
IGF-1
C2C12-IRS-1 KO/ GFP-mIRS-1
C2C12-IRS-1 KO/ GFP-mIRS-1
C2C12-IRS-1 KO/ GFP-mIRS-1ΔSAR
C2C12-IRS-1 KO/ GFP-mIRS-1 9YA
GFP-mIRS-1
GFP-mIRS-1ΔSAR
GFP-mIRS-1 9YA
Grb2
Merge
Relative fluorescence intensity
Distance (μm)
C2C12 IRS-1 KO/GFP-mIRS-1
C2C12 IRS-1 KO/GFP-mIRS-1ΔSAR
C2C12 IRS-1 KO/GFP-mIRS-1 9YA
Puncta co-localized with Grb2
IGF-1
e
Punctum diameter(μm)
p85
Grb2

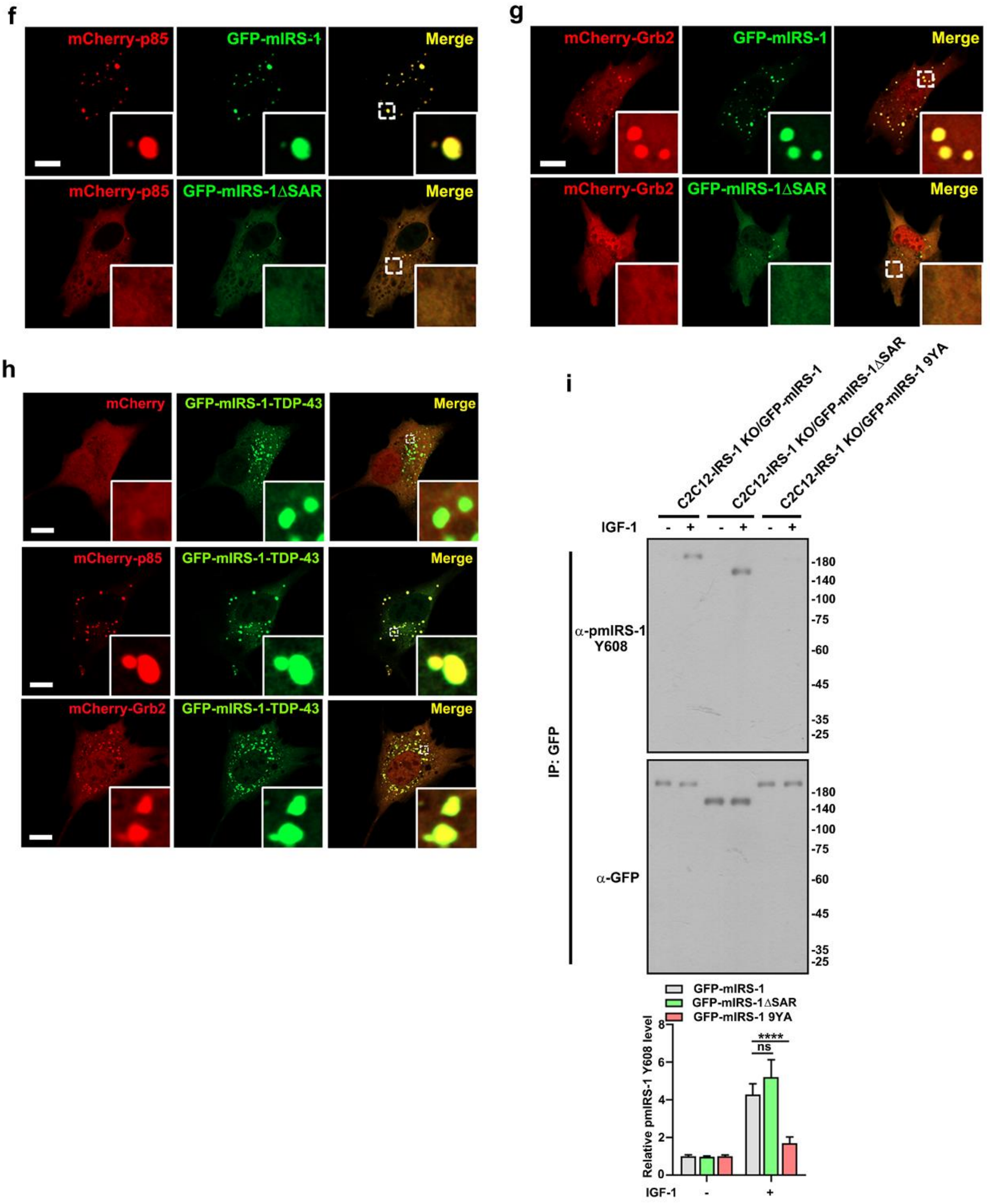
f
mCherry-p85
GFP-mIRS-1
Merge
mCherry-p85
GFP-mIRS-1ΔSAR
Merge
g
mCherry-Grb2
GFP-mIRS-1
Merge
mCherry-Grb2
GFP-mIRS-1ΔSAR
Merge
h
mCherry
GFP-mIRS-1-TDP-43
Merge
mCherry-p85
GFP-mIRS-1-TDP-43
Merge
mCherry-Grb2
GFP-mIRS-1-TDP-43
Merge
i
C2C12-IRS-1 KO/GFP-mIRS-1
C2C12-IRS-1 KO/GFP-mIRS-1ΔSAR
C2C12-IRS-1 KO/GFP-mIRS-1 9YA
IGF-1
- + - + - +
IP: GFP
α-pmIRS-1 Y608
α-GFP
-180
-140
-100
-75
-60
-45
-35
-25
GFP-mIRS-1
GFP-mIRS-1ΔSAR
GFP-mIRS-1 9YA
Relative pmIRS-1 Y608 level
8
6
4
2
0
****
ns
IGF-1
-
+

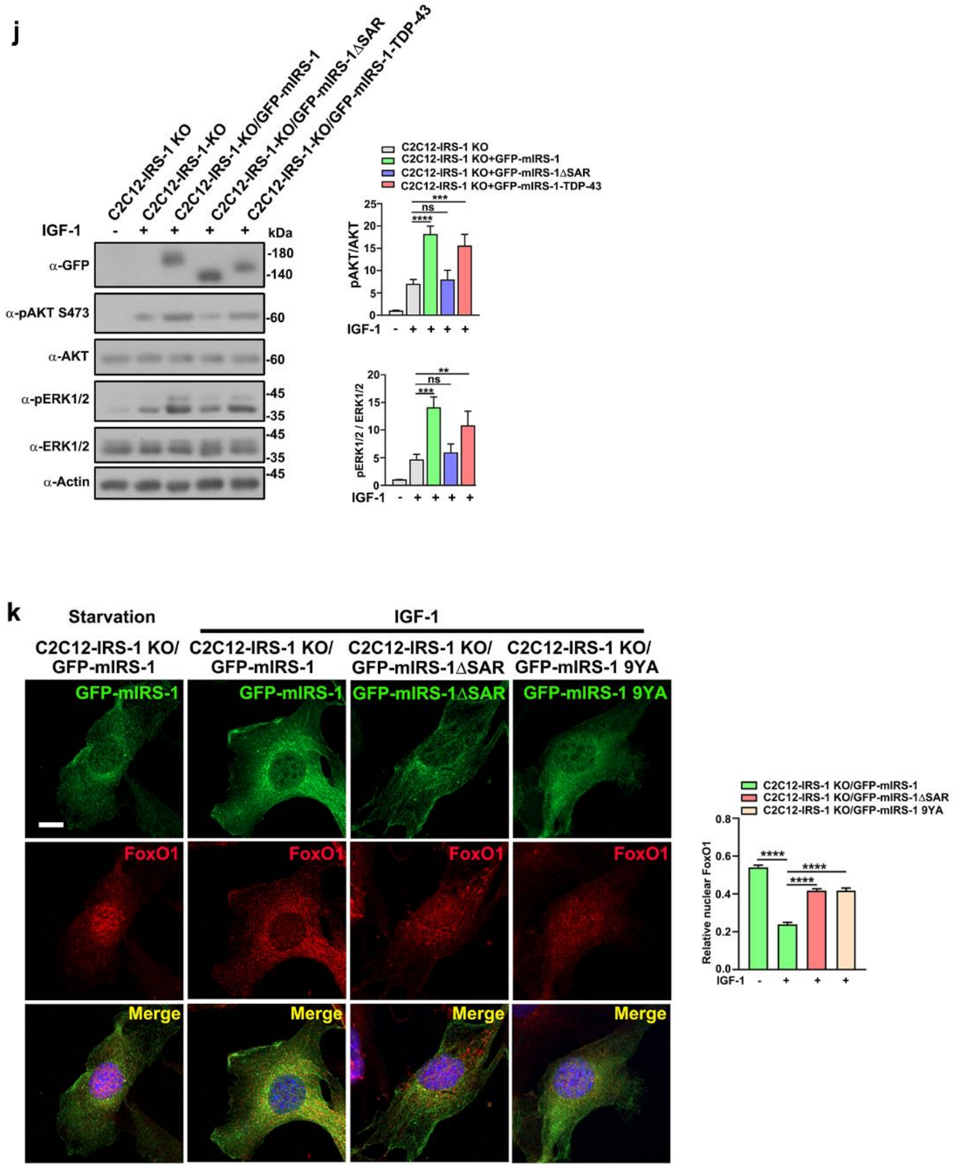

Fig. S5. IRS-1 recruits downstream effectors to form insulin/IGF-1 signalosomes.

a Immunoblot analysis of IRS-1 expression levels in C2C12 wildtype, C2C12/mCherry-p85 and C2C12-IRS-1 KO/mCherry-p85 cell lines. **b** Confocal image of mCherry-p85 foci in C2C12/mCherry-p85 cells treated with control or with IGF-1-conditioned (100 ng/ml) medium for 2.5 min. Scale bar, 10 μm. Quantitative analysis of number of mCherry-p85 puncta with results shown as violin plot. **: $p<0.01$. **c** Confocal image of mCherry-p85 foci in C2C12/mCherry-p85 cells treated with control

or with insulin-conditioned (100nM) medium for 2.5 min. Scale bar, 10 μm. Quantitative analysis of number of mCherry-p85 puncta is shown with the quantification result shown as violin plot. ***: p<0.001. **d** Confocal images of endogenous Grb2 and GFP-mIRS-1 or mutants in the indicated cell lines. Scale bar, 10 μm. Line scan shows the related intensity profiles of mIRS-1 with Grb2. The puncta co-localized with Grb2 were quantified (n=36). Data in the bar graphs represent the mean ± SEM. ****: p<0.0001. **e** The puncta diameter of GFP-mIRS-1 colocalized with endogenous p85 or Grb2 in **d** and **Fig. 5c** was quantified (n=80). Data in the bar graphs represent the mean ± SEM. **f** Confocal images of representative C2C12 cells co-expressing mCherry-p85 (red) and GFP-mIRS-1 or GFP-mIRS-1 ΔSAR mutant (green). Scale bar, 10 μm. **g** Confocal images of representative C2C12 cells co-expressing mCherry-Grb2 (red) and GFP-mIRS-1 or GFP-mIRS-1 ΔSAR mutant (green). Scale bar, 10 μm. **h** Confocal images of representative C2C12 cells co-expressing GFP-mIRS-1-TDP-43 mutant (green) and mCherry-p85 (red) or mCherry-Grb2. Scale bar, 10 μm. **i** GFP-tagged mIRS-1 wildtype or mutants were immunoprecipitated in IGF-1-stimulated or control C2C12-IRS-1 KO/GFP-mIRS-1, C2C12-IRS-1 KO/GFP-mIRS-1 ΔSAR or C2C12-IRS-1 KO/GFP-mIRS-1 9YA cell lines and then subjected to Western blot with p-mIRS-1 Y608 antibodies. Data in the bar graphs represent the mean ± SEM values of the ratios of densities for three independent experiments. ****: p<0.0001. ns: not significant. **j** Immunoblot analysis of total and phosphorylated AKT and ERK levels in C2C12-IRS1 KO, C2C12-IRS1 KO/GFP-mIRS-1, C2C12-IRS1 KO/GFP-mIRS-1 ΔSAR or C2C12-IRS1 KO/GFP-mIRS-1-TDP-43 cell lines treated

with or without IGF-1 conditional medium for 2.5 min. Data in the bar graphs represent the mean ± SEM values of the ratios of densities for three independent experiments. **: p<0.01. ***: p<0.001. ****: p<0.0001. ns: not significant. **k** Confocal images of endogenous FoxO1 in the starved or IGF-1-stimulated indicated cell lines. Data in the bar graphs represent the mean ± SEM values (n=50). ****: p<0.0001. Scale bar, 10 μm.

Sup. Fig. 6

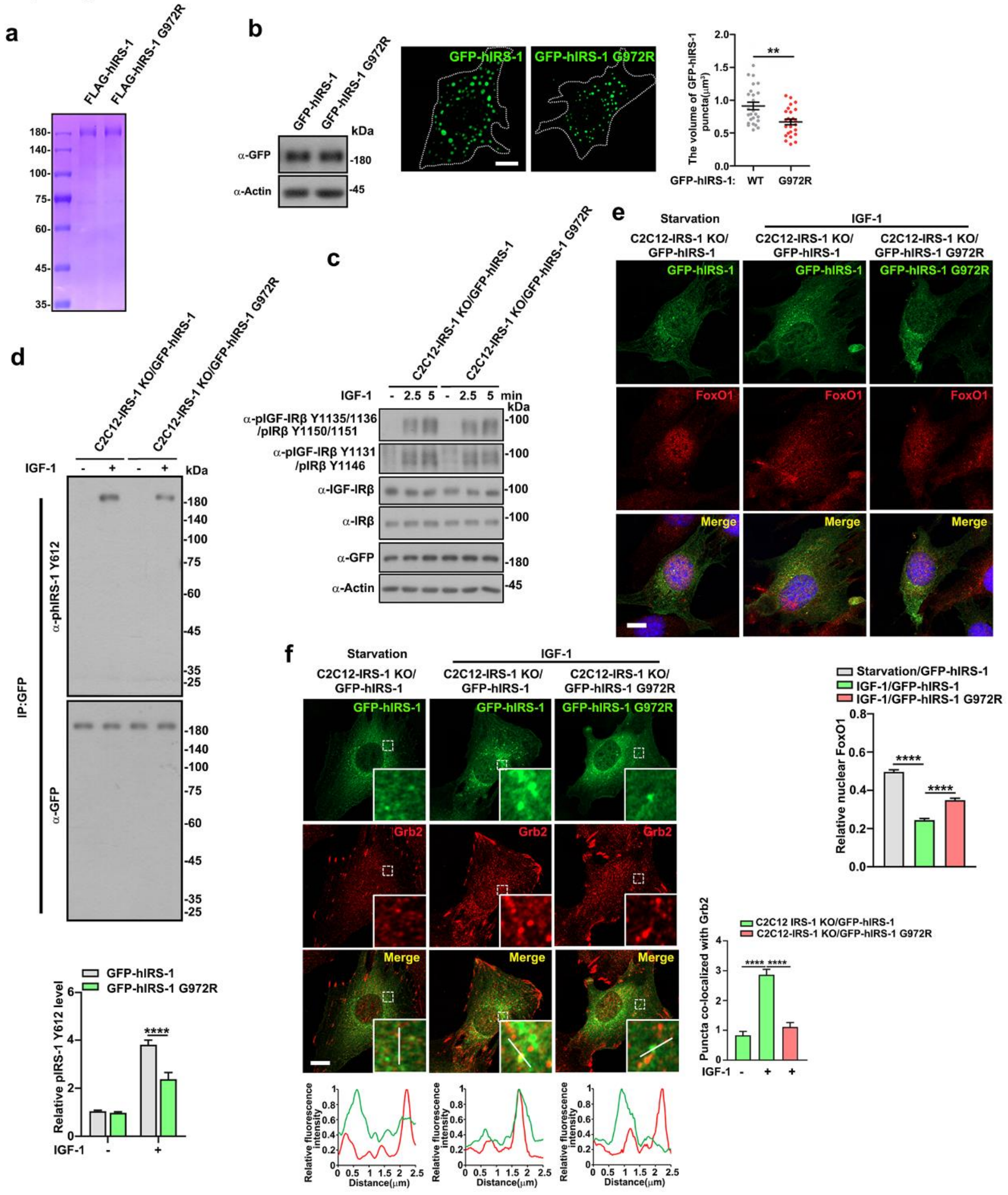

Fig. S6. The metabolic disease-related hIRS-1 G972R mutant undergoes altered phase transition. a SDS-PAGE and Coomassie blue staining results of purified recombinant FLAG-hIRS-1 and FLAG-hIRS-1 G972R proteins. **b** Confocal images of representative C2C12 myoblasts expressing GFP-hIRS-1 or GFP-IRS-1 hG972R mutant. Scale bar, 10 μm. Western blot analysis displaying the GFP-hIRS-1 and GFP-hIRS-1 G972R mutants as expressed at a similar level. Quantitative analysis of volume of GFP-hIRS-1 and GFP-hIRS-1 G972R mutant puncta is shown (n=26). **: p<0.01. **c** Immunoblot analysis of the indicated pIGF-1Rβ and pIRβ tyrosine phosphorylation levels in C2C12-IRS-1 KO/GFP-hIRS-1 and C2C12-IRS-1 KO/GFP-hIRS-1 G972R cell lines. **d** GFP-tagged hIRS-1 wildtype or mutants were immunoprecipitated in IGF-1-stimulated or control C2C12-IRS-1 KO/GFP-hIRS-1 and C2C12-IRS-1 KO/GFP-hIRS-1 G972R cell lines and then subjected to Western blot with p-mIRS-1 Y608 antibodies. Data in the bar graphs represent the mean ± SEM values of the ratios of densities for three independent experiments. ****: p<0.0001. **e** Confocal images of endogenous FoxO1 in the starved or IGF-1-stimulated C2C12-IRS-1 KO/GFP-hIRS-1 and C2C12-IRS-1 KO/GFP-hIRS-1 G972R cell lines. Data in the bar graphs represent the mean ± SEM values (n=46). ****: p<0.0001. Scale bar, 10 μm. **f** Confocal images of endogenous Grb2 and GFP-hIRS-1 or GFP-hIRS-1 G972R in the starved or IGF-1-stimulated C2C12-IRS-1 KO/GFP-hIRS-1 and C2C12-IRS-1 KO/GFP-hIRS-1 G972R cell lines. Scale bar, 10 μm. Line scans show the relative intensity profiles of hIRS-1 with Grb2. The GFP-hIRS-1 puncta co-localized with Grb2 were quantified (n=36). Data in the bar graphs represent the mean ± SEM. ****: p<0.0001.

Table S1: Plasmid information

Recombinant DNA	Vector backbones	Restriction enzyme cutting site
FLAG-mIRS-1	pXJ40-FLAG	Hind III and Not I
FLAG-mIRS-1 1-300 (PH-PTB)	pXJ40-FLAG	BamH I and Xma I
FLAG-mIRS-1 301-600	pXJ40-FLAG	BamH I and Xma I
FLAG-mIRS-1 601-800	pXJ40-FLAG	Hind III and Not I
FLAG-mIRS-1 801-1000	pXJ40-FLAG I	Hind III and Not I
FLAG-mIRS-1 1001-1233	pXJ40-FLAG	Hind III and Not I
FLAG-hIRS-1	pXJ40-FLAG	Hind III and Not I
FLAG-hIRS-1 G972R	pXJ40-FLAG	Hind III and Not I
GFP-mIRS-1	pXJ40-GFP	Hind III and Not I
GFP-mIRS-1 131-1233	pXJ40-GFP	Hind III and Not I
GFP-mIRS-1 301-1233 (IDR)	pXJ40-GFP	Hind III and Not I
GFP-mIRS-1 401-1233	pXJ40-GFP	Hind III and Not I
GFP-mIRS-1 501-1233	pXJ40-GFP	Hind III and Not I
GFP-mIRS-1 601-1233	pXJ40-GFP	Hind III and Not I
GFP-mIRS-1 701-1233	pXJ40-GFP	Hind III and Not I
GFP-mIRS-1 801-1233	pXJ40-GFP	Hind III and Not I
GFP-mIRS-1 1001-1233	pXJ40-GFP	Hind III and Not I
GFP-mIRS-1 Δ300-600 (SAR)	pXJ40-GFP	Hind III and Not I
GFP-mIRS-1 Δ600-800	pXJ40-GFP	Hind III and Not I
GFP-mIRS-1 Δ800-1000	pXJ40-GFP	Hind III and Not I
GFP-mIRS-1 Δ1001-1233	pXJ40-GFP	Hind III and Not I
GFP-mIRS-1 9YA	pXJ40-GFP	Hind III and Not I
GFP-hIRS-1	pXJ40-GFP	Hind III and Not I
GFP-hIRS-1 G972R	pXJ40-GFP	Hind III and Not I
mCherry-p85	pXJ40-mCherry	BamH I and Xma I
mCherry-Grb2	pXJ40-mCherry	BamH I and Xma I
RFP-Rab5	pXJ40-RFP	BamH I and Xho I
HP138-GFP-mIRS-1	HP138-puro (Addgene:134246)	N/A
HP138-GFP-mIRS-1Δ300-600 (SAR)	HP138-puro	N/A
HP138-GFP-mIRS-1 9YA	HP138-puro	N/A
HP138-GFP-hIRS-1	HP138-puro	N/A
HP138-GFP-hIRS-1 G972R	HP138-puro	N/A
HP138-GFP-mIRS-1 Δ600-800	HP138-puro	N/A
HP138-GFP-mIRS-1 Δ800-1000	HP138-puro	N/A
HP138-GFP-mIRS-1 Δ1001-1233	HP138-puro	N/A

HP138- mCherry-p85	HP138-puro	N/A
lentiCRISPRv2-gRNA	lentiCRISPRv2	BsmB I
